# Multi‐pronged analysis of pediatric low‐grade glioma and ganglioglioma reveals a unique tumor microenvironment associated with BRAF alterations

**DOI:** 10.1111/bpa.70023

**Published:** 2025-06-30

**Authors:** Shadi Zahedi, Kent Riemondy, Tian Liu, Andrea M. Griesinger, Andrew M. Donson, April A. Apfelbaum, Rui Fu, Julian Grandvallet Contreras, Michele Crespo, John DeSisto, Madeline M. Groat, Emil Bratbak, Adam Green, Todd C. Hankinson, Michael Handler, Rajeev Vibhakar, Nicholas Willard, Nicholas K. Foreman, Tzu Phang, Jean Mulcahy Levy

**Affiliations:** ^1^ Department of Pediatrics University of Colorado Anschutz Medical Campus Aurora Colorado USA; ^2^ Department of Pediatrics Morgan Adams Foundation Pediatric Brain Tumor Research Program, Children's Hospital Colorado Aurora Colorado USA; ^3^ Clinical Science Program, Graduate School, Anschutz Medical Campus University of Colorado Denver Colorado USA; ^4^ RNA Bioscience Initiative, University of Colorado Anschutz Medical Campus Aurora Colorado USA; ^5^ Department of Pediatric Oncology Broad Institute Dana‐Farber/Boston Children’s Cancer and Blood Disorders Center, Dana‐Farber Cancer/Boston Children's Cancer and Blood Disorders Center Boston Massachusetts USA; ^6^ Department of Pediatrics, Harvard Medical School Broad Institute of MIT and Harvard Cambridge Massachusetts USA; ^7^ Department of Neurosurgery University of Colorado Anschutz Medical Campus Aurora Colorado USA; ^8^ Department of Pathology University of Colorado Anschutz Medical Campus Aurora Colorado USA; ^9^ Department of Pharmacology University of Colorado Anschutz Medical Campus Aurora Colorado USA

**Keywords:** chemokine, ganglioglioma, Pilocytic astrocytoma, scRNA‐sequencing, spatial transcriptomics

## Abstract

Pediatric low‐grade gliomas (pLGG) comprise 35% of all brain tumors. Despite favorable survival, patients experience significant morbidity from disease and treatments. A deeper understanding of pLGG biology is essential to identify novel, more effective, and less toxic therapies. We utilized single‐cell RNA sequencing (scRNA‐seq), spatial transcriptomics, and cytokine analyses to characterize and understand tumor and immune cell heterogeneity of pilocytic astrocytoma (PA) and ganglioglioma (GG). scRNA‐seq revealed tumor and immune cells within the tumor microenvironment (TME). Tumor cell subsets include both progenitor and mature cell populations. Immune cells included myeloid and lymphocytic cells. There was a significant difference between the prevalence of two major myeloid subclusters between PA and GG. Bulk and single‐cell cytokine analyses evaluated the immune cell signaling cascade with distinct immune phenotypes among tumor samples. *KIAA1549‐BRAF* tumors appeared more immunogenic, secreting higher levels of immune cell activators and chemokines, compared to *BRAF V600E* tumors. Spatial transcriptomics revealed the differential gene expression of these chemokines and their location within the TME. A multi‐pronged analysis demonstrated the complexity of the PA and GG TME and differences between genetic drivers that may influence their response to immunotherapy. Further investigation of immune cell infiltration and tumor‐immune interactions is warranted.

## Graphical abstract

A multi pronged analysis approach revealed distinct neoplastic populations in PA and GG. Non‐neoplastic populations in PA showed higher levels of immune mobilizing chemokines in PA compared to GG.

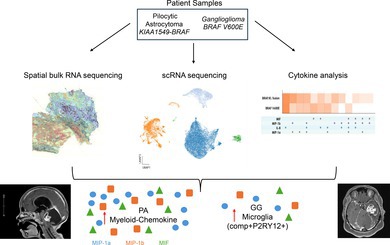

## 1 INTRODUCTION

Pediatric low‐grade gliomas (pLGG) are the most common type of pediatric brain tumors, accounting for approximately one third of all central nervous system (CNS) tumors [1]. Surgical resection remains the first line of therapy and can be curative in some cases. For the remainder of patients, chemotherapy and sometimes radiation are needed. Although the 5‐year event‐free survival is encouraging in historical studies, 40% of pLGG patients relapse within 5 years of diagnosis and require additional therapy [2]. Additionally, depending on the location of the tumor and the mutations they carry, pLGG can lead to significant neurologic damage, multiple recurring rounds of therapy, and, in some patients, ultimately death [3]. The significant morbidity and even mortality associated with pLGG highlight an urgent need for new treatment strategies.

Harnessing the immune system to treat human cancer has transformed the field of oncology but has yet to significantly impact the pLGG population. Immunotherapies have shown promising results in several tumor types. However, efficacies of such treatments vary across tumors and patients [4, 5]. CNS tumors have been particularly difficult to target. Recent data suggest the CNS is an immunologically ‘distinct’ location [6]. Despite this new understanding of how the immune system can interact with the CNS, studies have shown that brain tumors are able to develop immune evasion in a variety of unique ways such as sequestering T‐cells in the bone marrow to encourage antigenic ignorance [7]. Traditionally, CNS tumors were found to have low numbers of tumor‐infiltrating lymphocytes and other immune effector cells [8]. This is felt to be primarily related to the tumor/immune microenvironment in the CNS which has evolved to tightly regulate inflammation that could have detrimental effects in the enclosed cranial space [9]. Studies investigating immune infiltrating cells in adult glial tumors have uncovered tumor suppressive factors including myeloid‐derived suppressor and T‐regulatory cells, as well as immune suppressive metabolites. As a result, researchers have developed strategies to target inhibitory cells and molecules and utilize the power of the immune system to treat adult CNS tumors [10]. In previous studies from our group, we demonstrated different immune‐phenotypes across various pediatric brain tumor pathologies with a suggestion that pilocytic astrocytomas (PA) have a less suppressive immune environment [11]. Overall, studies on the nature of tumor infiltrating immune cells in pediatric pLGG are limited but may help with improving immunotherapy outcomes in these patients.

pLGG is thought of as a single pathway disease due to the uniform up‐regulation of the mitogen‐activated protein kinase (MAPK) pathway [12]. This is a result of mutations such as *BRAF V600E*, *NF1*, and *KIAA1549‐BRAF* fusion. *KIAA1549‐BRAF* fusion is the most frequent molecular alteration, identified in 30%–40% of pLGG [12]. This mutation is significantly enriched in PA and in tumors arising in the posterior fossa. PAs have highly circumscribed histology and normally arise in surgically amenable locations. However, when these tumors arise in deeply seated areas of the brain where complete surgical resection is not possible, progression becomes more common [12].

Other pediatric low‐grade tumor types carry a *BRAF V600E* mutation and vary notably depending on the histology and the location of the tumor. These include pleomorphic xanthoastrocytoma (40%–80%), diffuse astrocytoma (30%–40%) and ganglioglioma (GG) (25%–45%) [12]. GG are tumors composed of neoplastic ganglion and glial cells and are similar in grade to PA, both classified as a Grade 1. Compared to other pLGG, *BRAF V600E* tumors have the lowest overall and progression free survival. In the context of co‐occurring *CDKN2A* deletions, *BRAF V600E* tumors are significantly more likely to transform into higher‐grade tumors, often decades following the initial diagnosis [13].

Whole genome and RNA sequencing (RNA‐seq) technologies have been used to study gene expression patterns in many tumors. These studies identified additional mutations in *FGFR1*, *PTPN11*, and *NTRK2* fusion genes in pLGG [14]. In addition, the combination of RNA‐seq and copy number variation (CNV) data has identified novel fusion partners with the *BRAF* gene [15]. These data have brought a greater understanding of pLGG and ganglioglioma biology and advances to therapy including the use of MAPK pathway inhibitors. Although these inhibitors have decreased morbidity and improved survival, there is already evidence for developing resistance [16]. Additionally, these studies were performed on snap frozen, bulk tumor samples which lack the resolution to capture the complexity of the TME at the single cell level.

Single cell RNA‐seq (scRNA‐seq) provides the opportunity to investigate gene expression profiles at the single cell level and can provide insight into the diversity of tumor infiltrating immune cells. One scRNA‐seq study of six PA A2B5+ glial progenitor patient samples found these cells had a differentiated, astrocyte‐like phenotype and a smaller number of cells with a proliferative, progenitor‐like phenotype [17]. This study noted that 40% of identified cells were immune related, but there was insufficient expression of immune checkpoint genes, suggesting additional study of PA associated immune cells was needed. Most recently, a single‐nucleus RNA seq study evaluated five ganglioglioma patient samples and found CD34+ neuroectoderm‐like tumor precursor cells in addition to immune cells with myeloid and lymphoid lineage cells with potentially immune suppressive components [18]. These papers offer an early glimpse into the complex nature of PA and GG, but there remains a gap in understanding these tumors, the role of immune cells within them, and how these tumors compare to each other with neoplastic and non‐neoplastic components.

To attempt a broader understanding of PA and GG and the connected tumor microenvironment (TME) we investigated a panel of 23 patient samples using a multi‐pronged approach including scRNA seq, single cell and bulk cytokine analyses, and spatial transcriptomics (ST). Together, these data provide a clearer picture of a complex tumor heterogeneity between pLGG subtypes.

## 2 RESULTS

### 2.1 Patient sample characteristics

**FIGURE 1:**
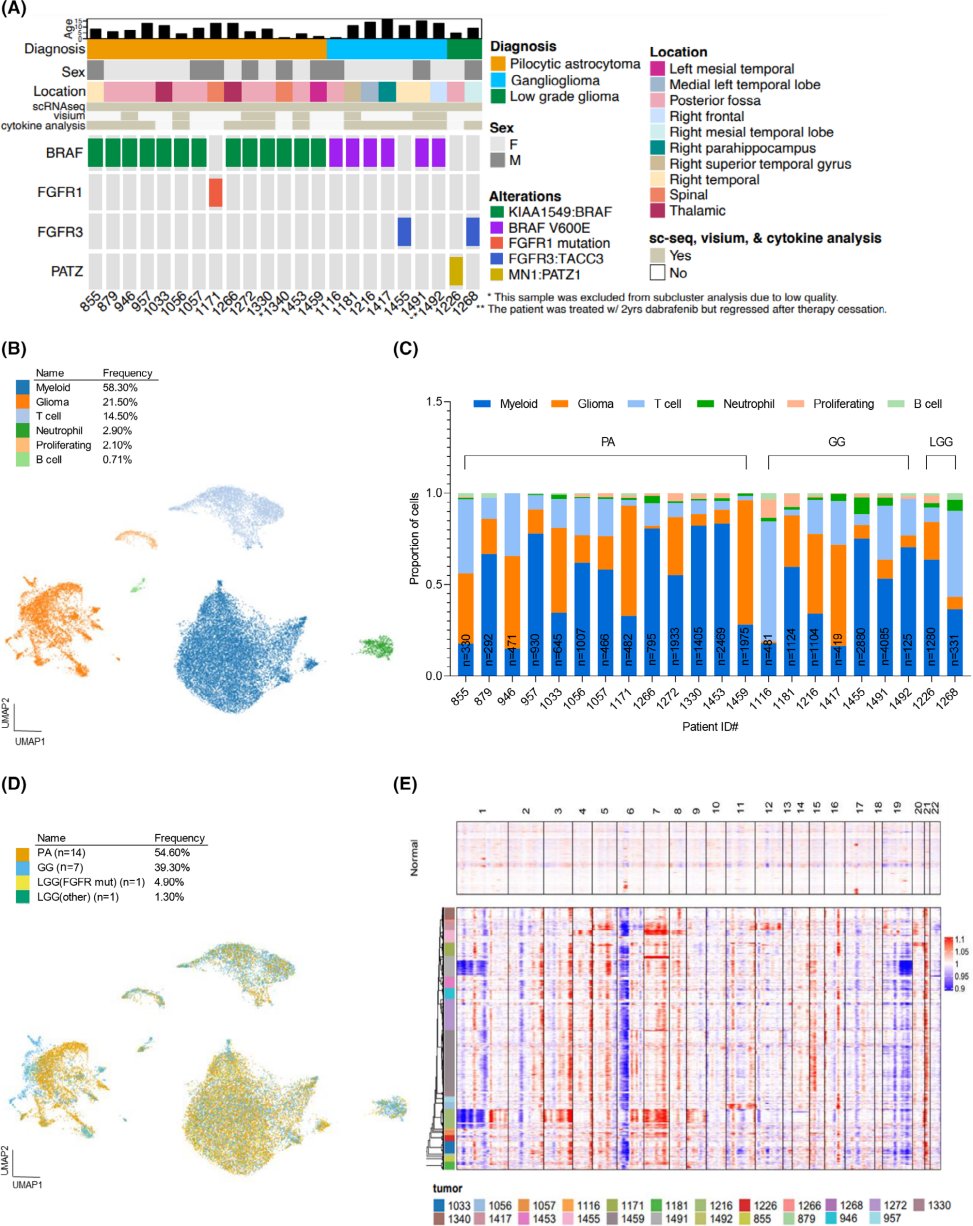
ScRNA‐seq analysis of pediatric low‐grade glioma reveals neoplastic and non‐neoplastic clusters. (A) Co‐mutation plot summarizing genomic alterations and clinical characteristics of 23 pLGG samples that underwent scRNA‐seq. Each column represents a patient's tumor and each row is a clinical, genomic, or other sample attribute. Different patient sample characteristics are shown by different colors. (B) Harmony aligned UMAP projection of single‐cell expression data of 23 pLGG samples colored by neoplastic and several non‐neoplastic (myeloid, T cell, B cell, Nt, proliferating) clusters. Cluster type abundance is indicated by %frequency in the legend. (C) Stacked bar charts show the contribution of each patient sample single cells to the neoplastic and non‐neoplastic clusters. Cell counts for each sample are normalized to 1 and are color‐coded according to the specific cluster. (D) Harmony aligned UMAP projection of neoplastic and non‐neoplastic clusters colored by tumor type. The number of samples and cellular abundance contributing to each tumor type is indicated in the legend. (E) Inference of CNV (inferCNV) profiles of neoplastic and non‐neoplastic PLGG single cells. CNV, copy number variants; GG, ganglioglioma; LGG, low‐grade glioma; mut, mutation; Nt, neutrophils; PA, pilocytic astrocytoma.

Following informed consent, samples were obtained from pLGG and GG patients who underwent tumor resection during 2011–2019 (Figure 1A, Supplementary Table 1). Samples were disaggregated at the time of surgery and viably banked at the Morgan Adams Pediatric Neuro‐Oncology Program Biobank. All samples were from initial diagnosis except one GG sample (UPN# 1492) which was from the first recurrence after therapy (the patient was treated with dabrafenib for 2 years). The median age at diagnosis was 10 years (range, 1–17). The most common diagnosis was PA (*n* = 14) followed by GG (*n* = 7) and not otherwise specified undefined low‐grade gliomas (*n* = 2). Tumor locations varied across samples, although the most common site was the posterior fossa. The majority of PA patients carried *KIAA1544:BRAF* fusion alteration (*n* = 13) and one PA had a *FGFR1* K565 and D562 mutation. Six of seven GG patients carried the *BRAF* V600E mutation and 1 patient carried a *FGFR3:TACC3* fusion mutation. One undefined LGG sample was found to have an *MN1:PATZ1* fusion, and the other carried a *FGFR3:TACC3* fusion. We confirmed the mutation status of all tumors by genetic profiling of bulk tissues.

### 2.2 scRNA‐seq of pLGG demonstrates neoplastic and non‐neoplastic subgroups

To determine the extent of cellular heterogeneity and complexity of pLGG, GG and their microenvironment, we performed scRNA‐seq on 23 primary patient samples (Figure 1). Cell Ranger (10X Genomics) and Seurat Analyses were used to filter and normalize cells, resulting in 26,029 cells that passed quality controls (Figure 1B and Supplementary Figure 1). These cells were projected as UMAP plots, revealing multiple clusters shared across different samples (Figure 1B, C). Harmony alignment was used to correct for inter‐sample variations from experimental or sequencing batch effects. Harmony alignment revealed multiple clusters harboring gene expression profiles of both neoplastic and non‐neoplastic clusters. Non‐neoplastic clusters comprised both myeloid and lymphoid lineage cells with myeloid cells comprising a higher proportion of cells (Figure 1B). As can be seen, there was a high frequency of myeloid cells (58.3%) compared to other cell types, specifically T cells (14.5%) (Figure 1B). To ensure this was not an artifact of process, we assessed three PA samples and a GG sample from the original patient group by IHC for myeloid (IBA‐1) and T cell (CD3) markers (Supplementary Figure 2). These demonstrated a similar increased presence of myeloid cells over T cells. Known neoplastic and non‐neoplastic markers were expressed in separate clusters as expected (Supplementary Figure 3). While there were differences in the proportion of neoplastic and non‐neoplastic cells in each patient sample, every individual sample included each cell type (Figure 1C). To examine the proportion of neoplastic and non‐neoplastic clusters identified by scRNA seq, we performed deconvolution analyses on bulk tissue transcriptome profiles using CYBERSORTx (Supplementary Figure 4). The data from bulk tissue demonstrated a similar finding of a mix of immune and tumor cells for each sample, although the proportion of glioma cells was higher in the deconvolution analysis.

Single cells were labeled by tumor type to assess the distribution of cells that comprise the different subtypes across major subclusters (Figure 1D). Non‐neoplastic cells from both PA and GG showed a similar distribution, although more GG cells were observed in T cell and Neutrophil clusters compared to PA cells. There were distinct differences in PA and GG tumor cell clusters (Figure 1D) suggesting a potential differential gene expression pattern.

We used CNVs to distinguish tumor cells from other cells. The most notable CNV events included losses on chromosomes 1, 6, and 19 as well as gains on chromosomes 3, 5, 7, and 21 (Figure 1E). For PA, inferred CNVs included events previously observed, including gains on chromosomes 5, 7, 15, and 20 [17, 19]. Additionally, we were able to detect BRAF V600E and BRAF:KIAA 1549 mutations in a subset of samples using scRNA and bulk RNA seq data (Supplementary Method). In summary, our scRNA‐seq data revealed both neoplastic and non‐neoplastic cells, with non‐neoplastic cells comprised of both myeloid and lymphocytic cell lineages.

### 2.3 Neoplastic population consists of distinct subpopulations in PA and GG

**FIGURE 2:**
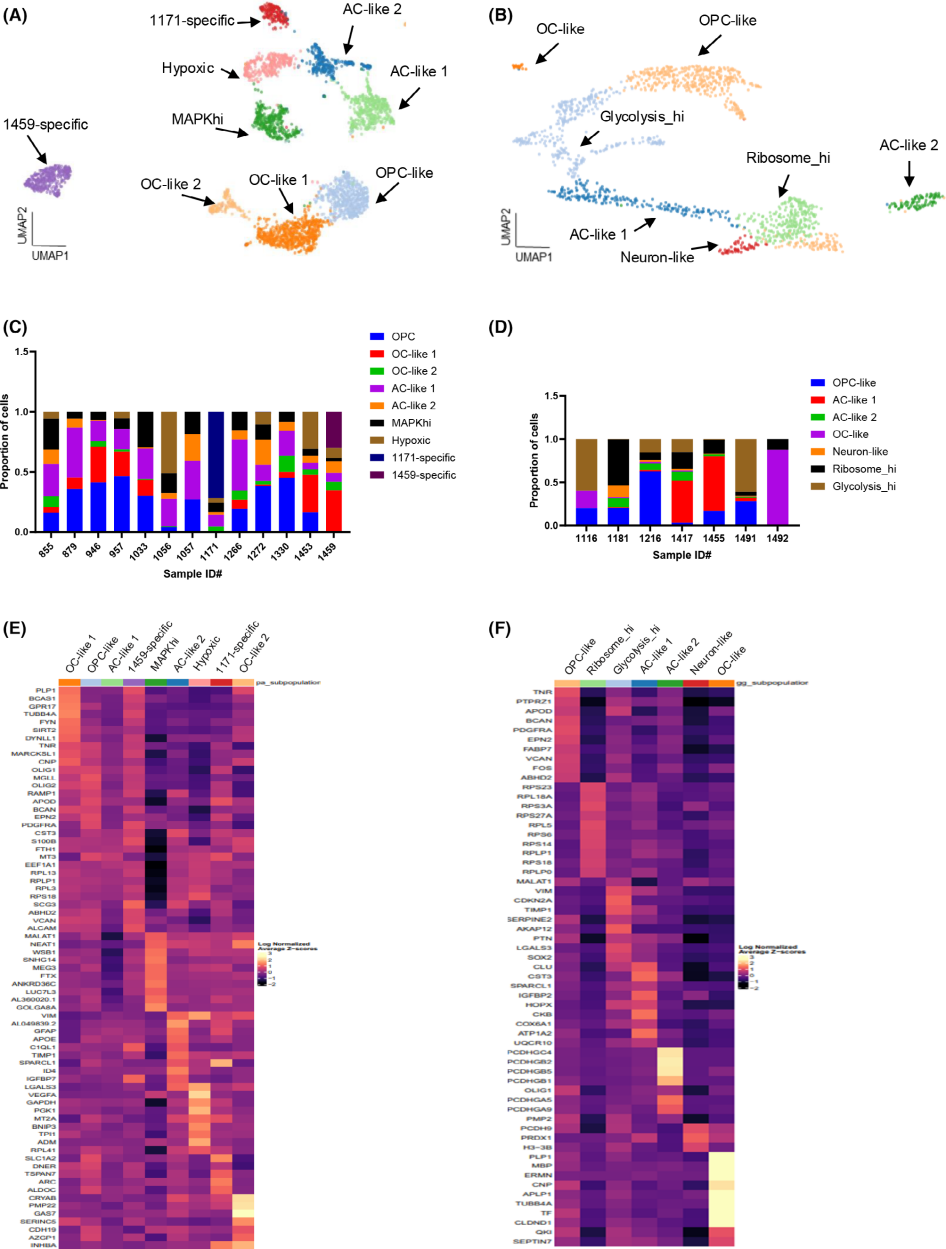
Gene expression profiling of neoplastic populations demonstrate distinct subpopulations in PA and GG. Harmony aligned UMAP plots of (A) PA cells colored by nine identified subclusters and (B) GG cells colored by seven identified subclusters. Stacked barcharts show the contribution of each patient sample to various (C) PA and (D) GG subclusters. Cell counts for subcluster are normalized to 1. Heatmaps for the top significant differentially expressed genes in various (E) PA and (F) GG subclusters. Each row represents a gene and each column represents a neoplastic cluster. AC, astrocytes; OC, oligodendrocytes; OPC, oligodendrocyte progenitor cells; MAPKhi, high expression levels of MAPK genes; Glycolysis_hi, high expression levels of genes involved in glycolysis; Ribosome_hi, high expression levels of ribosomal genes.

To assess differences in cellular identity between PA and GG, neoplastic cells were separated from non‐neoplastic cells and re‐clustered using Harmony. We identified nine PA (cell # = 3531) and seven GG subclusters (cell # = 1519) (Figure 2A, B). The number of cells comprising each neoplastic subcluster is detailed in Supplementary Table 2. Tumor and immune subclusters were labeled by comparing our dataset to external datasets using clustifyr [20]. Following further molecular characterization, we observed distinct cellular structures among PA and GG subpopulations. These subpopulations include cancer cells resembling oligodendrocyte progenitor cells (OPC‐like), oligodendrocytes (OC‐like), astrocytes (AC‐like), and specifically for GG, neurons (Neuron‐like). Additionally, we identified cells with high levels of MAPK genes specifically in PA. Furthermore, several less‐defined subpopulations, including cancer cells demonstrating hypoxic conditions (in PA) as well as cells with high levels of ribosomal and glycolysis activities (in GG) were identified. The contribution of each patient sample to various neoplastic subclusters is shown in Figure 2C, D.

The most abundant PA subcluster resembled OC (OC‐like 1). These cells appear as differentiating oligodendrocytes (Supplementary Figure 5A) differentially expressing genes including *PLP1* as well as *GPR17* and *BCAS1*. Studies have identified *PLP1* and BCAS1 as the most abundant myelin protein and a novel myelin‐associated protein, respectively. Interestingly, *BCAS1* has been shown as an OC‐like gene signature in a previous study investigating the transcriptional landscape of PAs [17, 21]. *GPR17* is a suggested regulator of oligodendrocyte development and specification [22]. There was a smaller OC‐like 2 population, which, although correlated with oligodendrocyte populations within the Allen Brain Atlas (Supplementary Figure 6A), appeared to have a unique gene expression profile compared to OC‐like 1. CRYAB and PMP22 are among the top differentially expressed genes within the OC‐like 2 cluster and have been shown to be myelin‐associated proteins [23, 24]. Finally, in PA, we identified a subcluster of cells showing high MAPK activity (Supplementary Figure 7). This subcluster differentially expresses genes enriched in the MAPK signaling pathway (MAPKhi) such as WSB1, SNHG14, and MEG3. These long noncoding RNAs have been shown to regulate the MAPK pathway [25]. The presence of these differentially expressed transcripts in our MAPK subcluster correlates with previous studies by Reitman et al. [17].

An OPC‐like population was the largest cell population in GG (Figure 2B) [26]. This was one of the shared subclusters between PA and GG. Although in both populations we identified known OPC markers (i.e., PDGFR and BCAN), the gene expression patterns differed. Heatmaps showing the top significant differentially expressed genes for each cluster (Figure 2E, F) highlight that each neoplastic cluster is defined by a clear set of genes. To further visualize the significance, magnitude, and direction of change for gene expression across these genes, we created bubble plots (Supplementary Figure 8).

In GG, there was also a small set of OC‐like cells resembling a more mature oligodendrocyte population differentially expressing mature OC‐related genes such as MBP and ERMN. Astrocyte (AC‐like 1–2) subpopulations were identified in both tumor types. Interestingly, all four AC‐like populations in PA and GG showed different gene expression patterns (Figure 2E, F). AC‐like 1 in PA demonstrated a less‐defined gene expression profile relative to other clusters but was correlated with two of the Allen Brain Atlas astrocyte populations (Supplementary Figure 6A); these cells appear to be undergoing a high degree of protein synthesis (Supplementary Figure 5A). The smaller AC‐like population in PA (AC‐like 2), showed differential expression of GFAP and VIM, two known intermediate filaments in astrocytes. AC‐like 1 in GG contained several members of COX genes which are involved in mitochondrial respiratory chain complexes metabolism (Supplementary Figure 5B). The second less populated astrocytic cluster (AC‐like 2) showed differential expression in several genes (PCDHGC4, PCDHGB1, PCDHGB2, PCDHGB5) encoding cell adhesion molecules (Supplementary Figure 5B) that regulate astrocyte‐neuron interaction. The remaining identified subclusters for each population are detailed in Supplementary Figure 5.

There were two sample‐specific clusters (UPN#1459 and UPN#1171). In GG, there were cell populations that we could not clearly define, one carrying high ribosomal genes (Ribosome_hi) and one with high levels of glycolytic activity (Glycolysis_hi).

We also performed single‐cell RNA sequencing (RNAseq) gene set enrichment (GSEA) pathway analysis to compare the activity score of enriched pathways among different subclusters in PA and GG (Supplementary Figure 9). This data further supported the classification of our neoplastic clusters.

In summary, we observed several neoplastic subclusters in both PA and GG. Interestingly, some clusters identified having the same origin had different gene expression patterns.

### 2.4 Characterizing immune populations in PA and GG

**FIGURE 3:**
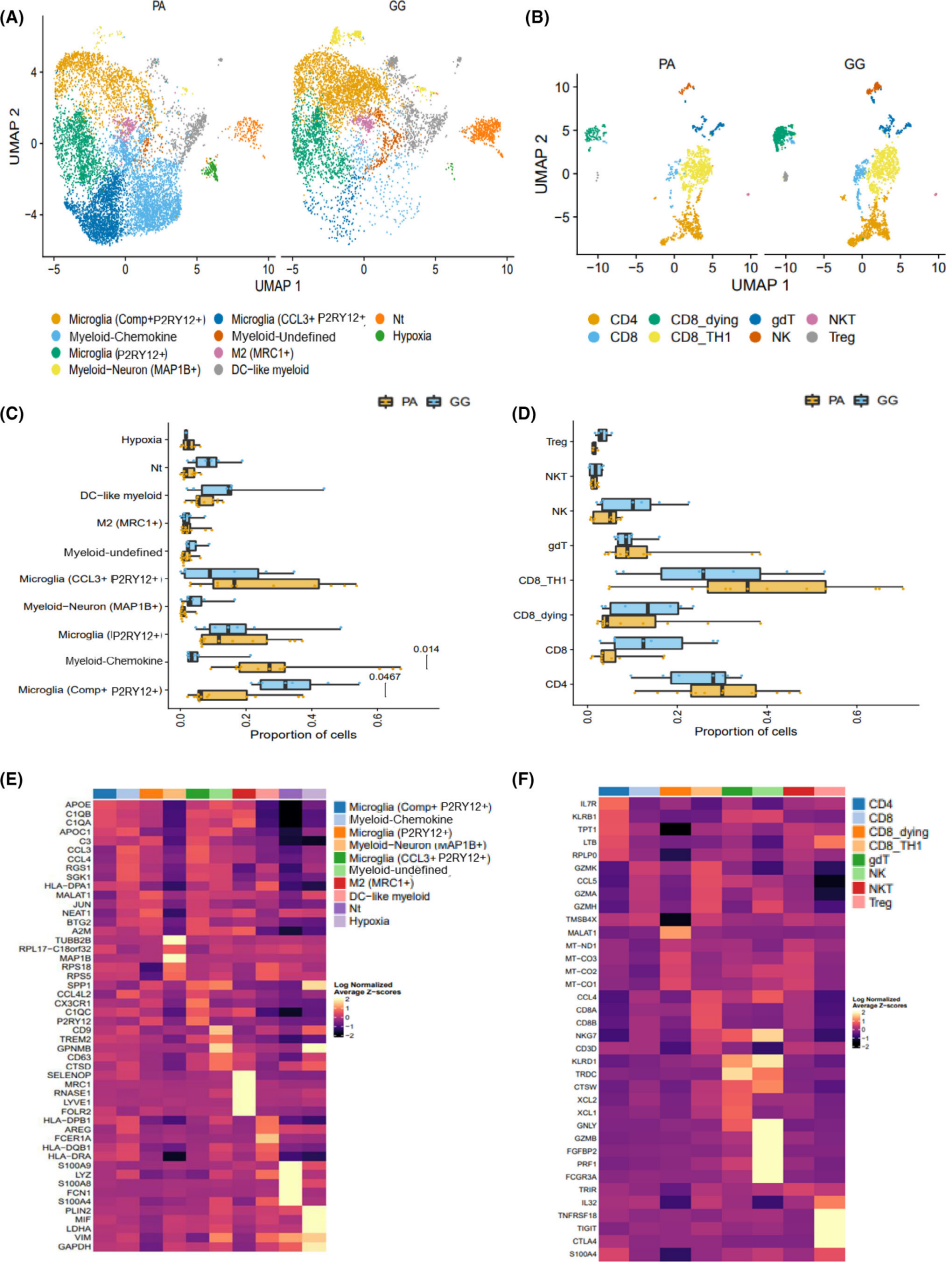
ScRNA‐seq analyses of immune cells in PA and GG. Harmony aligned UMAP plots of (A) myeloid and (B) T cell subclusters. Bargraphs comparing cell proportions in (C) myeloid and (D) T cell populations between PA and GG. Heatmaps for the top significant differentially expressed genes in various (E) myeloid and (F) T cell subclusters. Each row represents a gene and each column represents a non‐neoplastic cluster. DC, dendritic cells; GG, ganglioglioma; gdt, gamma delta T cells; NKT, natural killer T cells; NK, natural killer cells; Nt, neutrophils; PA, pilocytic astrocytoma; Treg, regulatory T cells.

To fully characterize immune subclusters, we separated the non‐neoplastic subclusters into myeloid and lymphoid lineages. Unsupervised clustering of each using Harmony revealed 10 myeloid lineage and eight lymphoid lineage subclusters (Figure 3A, B). The number of cells comprising each immune subpopulation is detailed in Supplementary Table 3. We identified three microglia populations differentially expressing P2RY12, a known marker of microglia (Microglia Comp+P2RY12), Microglia P2RY12+, and Microglia CCL3 + P2RY12. Microglia CCL3 + P2RY12 appeared to be functionally more activated, expressing high levels of chemokines such as *CCL3* and *CCL4*. Additionally, we identified a highly chemotactic myeloid subpopulation (Myeloid‐Chemokine), which also expressed chemokine molecules *CCL3* and *CCL4*. Among smaller clusters, we identified a myeloid‐tumor mix population (Myeloid‐Tumor). These cells appeared to most likely be low‐quality glioma cells. However, they were difficult to confidently assign as they tend to have very few genes and UMIs detected (~200–500). The markers for this population are largely expressed only in glioma cells (e.g., NFIB, MAP1B, TUBB2B) and they express low levels or have no expression of myeloid/immune markers (CD74, APOE, HLA genes, and CD45). We also detected a very small subset of cells differentially expressing genes associated with hypoxia (*MIF* and *LDHA*). All samples contributed to all myeloid subclusters to various degrees (Supplementary Figure 10A). We have mainly used non‐conventional identities to annotate myeloid subclusters, as myeloid and lymphoid cells shift away from the conventional identities in the presence of the tumor microenvironment [27, 28].

We compared the proportion of cells in myeloid subclusters between PA and GG samples (Figure 3C, D). While there appeared to be differences in cell proportions among all myeloid subclusters, we noticed a significant difference in cell proportion, particularly in two subclusters. Cells comprising the Myeloid‐Chemokine cluster were significantly more prevalent in PA compared to GG (*p* = 0.014). In comparison, Microglia (Comp+ P2RY12+) had a significantly higher prevalence in GG (*p* = 0.0467).

Unlike in the myeloid subclusters, there was no significant difference among T cell subclusters between PA and GG (Figure 3D). All patient samples contributed to the different T subclusters (Supplementary Figure 10B). Harmony clustering identified several T cell subclusters (Figure 3B) as well as smaller subsets of NK and NKT cells. Heatmaps showing the top differentially expressed genes for each cluster (Figure 3E, F) highlight that each non‐neoplastic cluster is also defined by a clear set of genes. We further visualized the significance, magnitude, and direction of change for gene expression across these genes using bubble plots (Supplementary Figure 11).

Our data revealed several myeloid and T cell subclusters that contributed to PA and GG tumor microenvironments. Interestingly, a significant difference was seen between the prevalence of major myeloid subclusters in PA and GG. To gain a better understanding of this underlying difference, we decided to study the immune signals via which these cells communicate by analyzing their secreted cytokines.

### 2.5 *KIAA‐1549:BRAF* fusion tumors appear to have higher levels of immune mobilizing cytokines compared to BRAF V600E tumors

**FIGURE 4:**
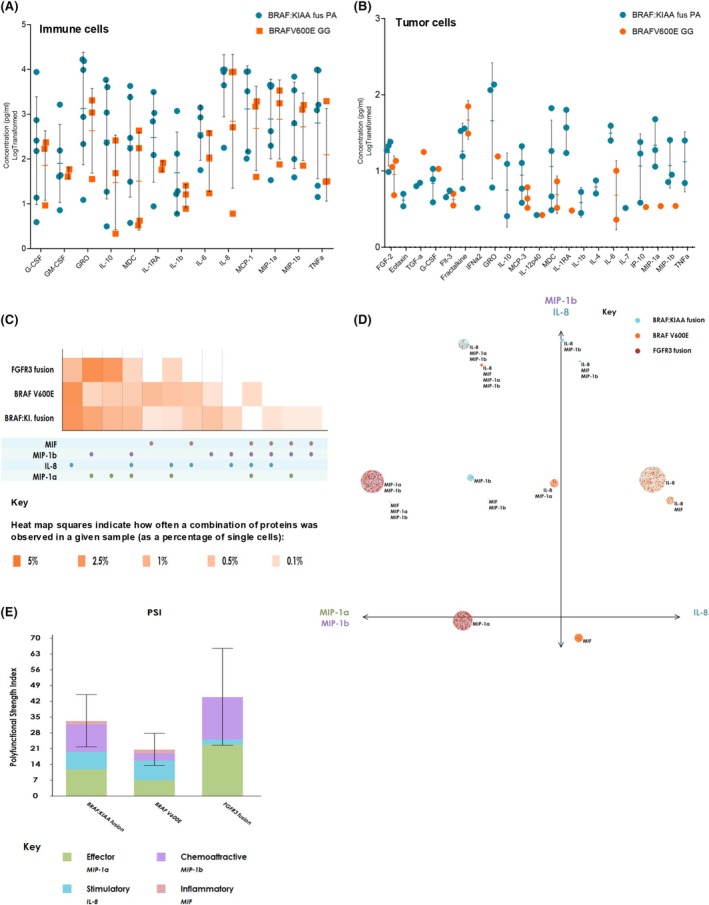
Bulk and single cell cytokine/chemokine analyses in PA and GG. Dot plots of detected cytokines/chemokines from bulk (A) myeloid (cytokines/chemokines with concentrations greater than 1000 pg/mL) and (B) tumor (all cytokines/chemokines) cells conditioned media in PA and GG. (C) Single cell polyfunctional heatmap displaying functional cytokines/chemokines of interest secreted across myeloid cells from samples PA and GG samples with different mutational status. The heatmap is color‐coded from light to dark, based on the frequency of the polyfunctional subsets. The rows of squares correspond to the two sample groups and the columns correspond to polyfunctional group of cytokines that was expressed in at least one of the sample groups. (D) Polyfunctional activity topography principle component analysis (PAT PCA) of particular functional groups across samples with different mutational status. Each color coded dot represented a single cell from one of our two sample groups. The size of the circles corresponds to the frequency of an individual polyfunctional group (large groups = large circles, small groups = small circles), and the cytokines labeled next to it represent the cytokines present in the functional group. The principle components are labeled according to their correlation with specific cytokines. PC1 and PC2 are a combination of dominant cytokines that drive the polyfunctionality of myeloid cells. (E) Polyfunctional strength index (PSI) computed for myeloid cells at the single cell level across two sample groups with different mutational statuses.

scRNA sequencing analysis of our immune populations led us to hypothesize that there would be differences in the cytokine signaling from myeloid cell populations between PAs and GGs and across BRAF mutational profiles. To investigate this, we performed a broad cytokine analysis utilizing two platforms to gain a better understanding of the function that each myeloid cell subpopulation has within pLGG. We performed bulk cytokine analysis on media collected from tumor and immune cells following a short culture and compared the secretion profile between *BRAF* V600E and *KIAA‐1549:BRAF* fusion samples. Each dot is representative of a patient sample run on the conditioned media multiplex cytokine/chemokine assay. We ran six *KIAA‐1549:BRAF* PAs and three *BRAF V600E*. Though not significant, there was a trend toward higher concentrations of cytokines secreted by KIAA‐1549:BRAF fusion samples for both tumor and immune cells (Figure 4A, B, Supplementary Figure 12).

To get a better understanding of the cytokine signaling in PA and GG myeloid cells, we performed single cell cytokine analyses. To distinguish distinct polyfunctional myeloid subsets and the heterogeneity within our patient samples, we utilized a polyfunctional heatmap (Figure 4C). This visualization shows major functional subsets secreted across different mutational groups (*KIAA‐1549:BRAF* fusion and *BRAF V600E*). As seen in Figure 4C, there is heterogeneity in polyfunctional groups across all sample groups. Overall, *KIAA‐1549:BRAF* fusion samples appear to have the most polyfunctional groups. It is interesting to note that there is a higher percentage of single cells in the *KIAA‐1549:BRAF* fusion group compared to the *BRAF V600E* group that express a combination of chemokines, MIP‐1*α* (CCL3) and MIP‐1*β* (CCL4) as well as a combination of MIP‐1*α*, MIP‐1*β*, and IL‐8 (CXCL8). A higher percentage of single cells in the *BRAF V600E* group express the pro‐tumor cytokine MIF, but this expression pattern changes when MIF is expressed along with the above‐mentioned chemokines.

To visualize distinct polyfunctional myeloid subsets and their complex landscape, we performed a modified PCA analysis called Polyfunctional Activation Topology Principle Component Analysis (PAT PCA). In the resulting scatter plot (Figure 4D), each functional group discussed in the heatmap is represented by a circle in the PAT PCA graph. Several functional groups are identified, each secreting a particular combination of cytokines. Consistent with what we identified in the polyfunctional heatmap, the PAT PCA graph also shows higher levels of MIP‐1*α* and MIP‐1*β* dominated polyfunctional groups secreted by *KIAA‐1549: BRAF* fusion cells compared to *BRAF V600E* single cells. Our data show that there are distinct populations of cells that are secreting one or more cytokines/chemokines across mutational subgroups. BRAF fusion cells appear to be in a more immunologically active state. In summary, these data highlight a functional difference between *KIAA‐1549: BRAF* fusion and *BRAF V600E* immune cells.

Finally, to quantify the collective impact of polyfunctional cells (simultaneously secreting multiple cytokines at high intensity), we used a polyfunctional strength index (PSI). PSI is defined as the percentage of polyfunctional cells within a particular sample multiplied by the average signal intensity of the cytokines that are secreted from those cells. The PSI revealed that chemokines including MIP‐1*α* and MIP‐1*β* have a higher impact in BRAF fusion tumors compared to *BRAF V600E* tumors (Figure 4E).

When you look at a CellChat analysis for the CCL pathway that incorporates MIP‐1*α* and MIP‐1*β* in the PA samples, the OC‐like‐2 cluster is the most prominent contributor to signaling toward the microglia population with a small component of OC‐like‐1 (Supplemental Figure 13). For the MIF pathway, the CellChat signaling is overall more complex. In PA, almost all major tumor subclusters contribute to signaling toward most myeloid subclusters, but Myeloid‐Chemokine and Dendritic‐like cell subclusters appear to have the strongest communication with tumor cells. All major tumor subclusters communicate with all major myeloid subclusters in GG. Interestingly, the AC‐like 2 population shows the weakest interaction with the immune subclusters (Supplemental Figure 13).

Collectively, these data suggest a complex and distinct immune phenotype between *KIAA1549‐BRAF* fusion and *BRAF V600E*, suggesting the involvement of potentially different immune pathways in these tumor subtypes.

### 2.6 Spatial transcriptomics (ST) reveal differential expression of chemokines in PA and GG

**FIGURE 5:**
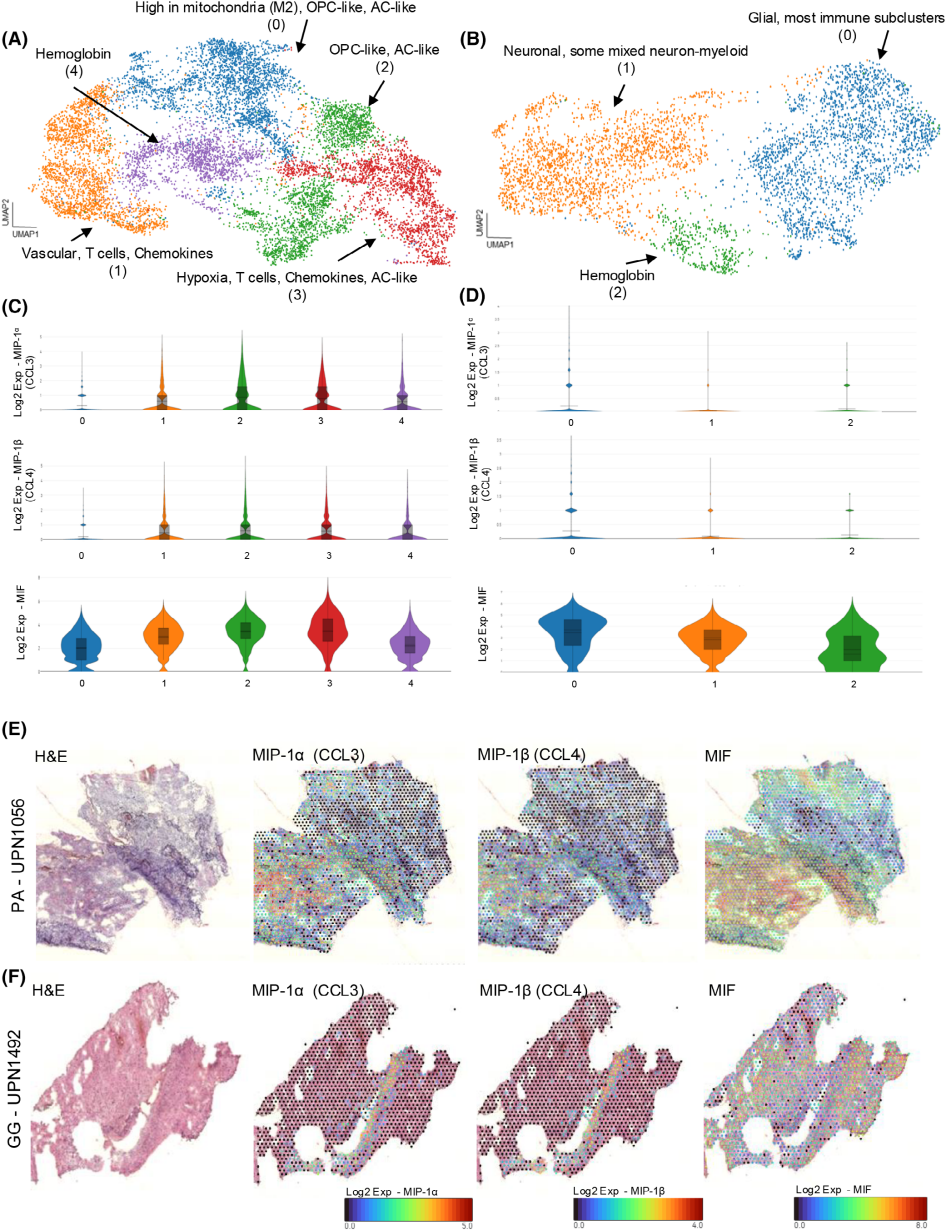
Cytokines/chemokines have a differential distribution within TME in PA and GG. UMAP clustering of spatial transcriptomic data colored by different subclusters in (A) PA and (B) GG. Each subcluster comprises of different neoplastic and non‐neoplastic cell types. Violin plots showing the expression levels of MIP‐1*α*, MIP‐1*β*, and MIF in (C) PA and (D) GG. Representative images showing spatial expression levels of MIP‐1*α*, MIP‐1*β*, and MIF in a (E) PA and (F) GG sample.

Both scRNA‐seq and single cell cytokine assays revealed differences in immune cell infiltration and function between PA and GG. However, single cell techniques lack spatial orientation of cells within the TME. Therefore, we performed ST analysis in both PA and GG using the Visium platform (10X genomics). This analysis was performed on five PA (UPN#946, UPN#1056, UPN#1272, UPN#1330, UPN#1453) and three GG (UPN#1181, UPN#1491, UPN#1492) snap frozen surgical sections. All PA and GG harbored BRAF fusion or BRAF V600E alterations, respectively. ST data was processed and filtered resulting in (total number of spots =14,990 (10,477 spots per PA and 4513 per GG samples)) across all samples. Sample spots were clustered with batch correction using harmony resulting in five PA and three GG clusters across the eight samples (Figure 5A, B).

Transcriptomic profiles from each spot cluster were examined using clustree analysis of ST cluster similarity and ontological analysis of ST marker genes. Different clustering resolutions were utilized for identifying cell types present in various regions of clusters. ST showed distinct regions within PA and GG tumors consisting of both immune and tumor cell populations (Figure 5A, B). Marker genes identifying cell types within various regions of PA and GG are shown in Supplementary Tables 4 and 5 respectively. As predicted by our cytokine data, higher expression of genes associated with identified cytokines (MIB‐1*α*, MIP‐1*β*, and MIF) was observed in PA compared to GG samples (Figure 5C–F, Supplementary Figure 14). This also supports higher signaling for these noted in the CellChat analysis (Supplementary Figure 13). These data support our cytokine analysis, indicating an importance and difference in cytokine gene expression between pLGG groups.

## 3 DISCUSSION

pLGG are a heterogeneous set of tumors with varying clinical behavior and varying responses to therapy. To help gain a deeper understanding of the heterogeneity of the tumor microenvironment and the potential role immune cells may play in pLGG, we used a three pronged approach with [1] scRNA‐seq to biologically characterize two of the most common pLGG subtypes PA and GG [2] bulk and single cell cytokine analysis for a more functional understanding of these tumors, and [21] ST analyses as a validation method for findings from our scRNA‐seq and cytokine analyses. We believe such a multi‐pronged approach comparing different tumor subtypes will be needed to fully understand how to develop successful immunotherapy for pLGG.

Previous data evaluating small populations of PA and GG assessed the tumor types independently [17, 18]. Our data allowed us to compare tumor subpopulations among our PA and GG tumor samples. Our results demonstrate bothimmature progenitor cells and mature oligodendrocytes and astrocytes. When compared with the previous PA study, our MAPKhi population showed the strongest correlation with their MAPKhi population. There was no substantial overlap between our OC‐like and AC‐like populations and theirs. This could be due to different methods of scRNA seq analysis resulting in differential capture of markers and limited sample size. Analysis of GG tumors using single nuclei RNA seq provided a comprehensive review of the transcriptomic landscape of these samples and a potential prognostic signature for this tumor type [18]. Their data would support our findings that GG may be less immunogenic and therefore less responsive to immune therapies.

Our scRNA‐seq analyses revealed several immune populations. The most notable observation was the identification of a large myeloid subpopulation in our overall pLGG dataset, comprising over 50% of cells. We identified several myeloid subclusters and observed a significant difference in two distinct subclusters (chemokine‐myeloid and microglia (comp+ P2RY12+)) between PA and GG. There were a significantly higher number of cells comprising the chemokine‐myeloid subcluster in PA compared to GG and a larger microglia (comp+ P2RY12+) subcluster in GG. The chemokine‐myeloid subcluster demonstrated an increased expression of genes responsible for the immune mobilizing chemokines MIP‐1*α* and MIP‐1*β*. Since chemokines are originally known to recruit other immune cells to the tissue site during both homeostasis and in response to infection and inflammation, this observation may suggest a higher level of immune activity in PA compared to GG.

Our scRNA seq data suggested cytokine signaling may be important to the overall immune function of these tumors. To evaluate the potential cytokine differences and examine the level of immune activity in our PA and GG samples, we performed bulk and single cell cytokine analyses. We observed specific chemokines such as MIP‐1*α* and MIP‐1*β* to be secreted in higher concentrations in fusion samples compared to BRAF V600E samples. This agrees with our scRNA‐seq analysis data. It has been shown that MIP‐1*α* plays a critical role in recruiting distinct immune phenotypes to intratumoral sites and it induces antigen specific T cell responses [29]. Our work has implications for vaccine trials aiming to utilize the power of the immune system against PA and GG [30]. The functional cytokine differences identified in our data suggest immunotherapy approaches toward tumors with *KIAA1548‐BRAF* fusion and *BRAF V600E* mutations may need to be customized for each group to capitalize and compensate for innate cytokine differences between them. Future studies will investigate the cytokine profiles from T cell populations isolated from our patient samples to better understand the functional classes of T lymphocytes, including exhausted T cells, which may help us assess the downstream impact of elevated chemokines and cytokines in PA tumors. To see if we can observe the differential expression of chemokines and cytokines within the tumor cellular architecture in intact tissues, we utilized spatial transcriptomics. Our data showed higher expression of chemokines of interest in the spatial environment of PA tumors compared to GG tumors. This was consistent with our scRNA seq and the cytokine data.

As with all studies, our analysis carries some limitations. Though four PA and GG samples (#1057, #1491, #1453, and #1455) contributed the most cells to the patient sample cohort of 23, two carry BRAF V600E, and two carry BRAF fusion mutations suggesting an equal representation of mutant subgroups. A larger sample size (especially for GG subtype) resulting in a higher number of cells could provide a clearer picture of the cellular identity of our immune and tumor subclusters. Additionally, future studies of lineage analyses will be needed for a deeper understanding of the identified subclusters. Additional future studies will also focus on how not only the genetic drives of tumors, but also how the location of tumors may influence these results. Finally, future additional pathway and functional analysis will be performed to better understand the downstream impact of elevated chemokines and cytokines in our PA fusion samples and to gain a broader understanding of the biology behind and affected by these processes.

Despite some of these limitations, we believe our study has expanded the critically important genomic and functional understanding needed for the role immune cells may play in the TME in the context of pLGG. In a recent study, authors investigated TME in a wide array of pediatric brain tumors, including pLGG. They demonstrated the critical importance of TME characterization alongside the driver mutation and the tumor mutation burden in how individual tumors respond to immunotherapy [31]. In pLGG, authors found that tumors with high tumor inflammation signature indicating a high immune cell infiltration such as BRAF mutant tumors, but not BRAF fusion tumors show the worst clinical outcome. Our data has revealed that pLGG contains a vast immune population, but there are differences between tumor types and genetic changes that may influence their response to immunotherapy. Our studies indicate PA tumors that carry the BRAF fusion mutation appear to have higher immune activity. Future functional and preclinical studies are underway to validate and complement our current studies. This comprehensive approach helps further our understanding of the potential impact of molecular differences on tumor biology and paves the way for developing potential immunotherapies for pLGG patients. Further investigation of the immune cell infiltration and tumor‐immune interactions is warranted.

## 4 MATERIALS AND METHODS

### 4.1 Sex as a biological variable

Our study examined male and female patient samples, and similar findings are reported for both sexes.

### 4.2 Patient sample acquisition and preparation

Human pLGG samples (*n* = 23) were collected from surgeries at Children's Hospital Colorado with IRB approval (COM‐IRB 95‐500) (Supplementary Table 1). For single cell approaches, tissue was collected in serum‐free media, brought to the laboratory for mechanical dissociation, and viably frozen in single cell suspension as previously described [32]. Samples utilized for spatial transcriptomics and cytokine analyses were snap frozen at surgery.

### 4.3 ScRNA‐seq analysis

scRNA‐seq was performed on 23 pLGG patient samples. Prior to sequencing, samples were thawed in batches and flow sorted (Astrios EQ) to obtain viable single cells based on propidium iodide (PI) exclusion. With the study goal of performing scRNA‐seq on 2000 cells per sample, we utilized a Chromium Controller in combination with Chromium Single Cell V3 Chemistry Library Kits, Gel Bead & Multiplex Kit and Chip Kit (10X Genomics). Single cells were isolated into microfluidic droplets containing oligonucleotide‐covered gel beads that capture and barcode the transcripts. Transcripts were converted to cDNA, barcoded and sequenced on Illumina NovaSeq 6000 sequencer to obtain approximately 50,000 reads per cell.

### 4.4 scRNA‐seq data analysis

Raw sequencing reads were processed into gene‐expression matrices using Alevin (salmon version 1.3.0) with the hg38 human reference genome. The resulting count matrices were further filtered in Seurat (v.4.0.3‐4.1.1) (https://satijalab.org/seurat/) to remove cell barcodes with less than 200 genes, more than 20% of UMIs derived from mitochondrial genes, or less than 500 UMIs and more than 50,000 UMIs. UMI counts were log‐normalized. PCA was performed on scaled normalized counts using highly variable genes (2000). Harmony (v0.1.0) was applied to integrate samples (theta = 1). A total of 25 harmony components were used for generating a UMAP projection and a shared nearest neighbor graph, which was clustered using graph based clustering implemented in Seurat. Doublet cells were identified using scDblFinder, and cells with doublet scores greater than 5 were excluded (v. 1.6.0). Cells were classified into broad cell types (T/NK, B, Myeloid, Neutrophil, Glioma, and Proliferating) by comparing clusters to a single cell reference dataset using clustifyr (v1.4.0) [20]. The custom reference was constructed using single cell RNA‐seq from other pediatric brain tumors and normal human cell datasets [21, 32, 33, 34, 35, 36]. In addition to automated cell type identification, tumor and stromal cell populations were further refined manually through comparisons of top cluster marker genes to markers of known tumor and stromal populations. Tumor cells from individual subgroups, and immune cell types (T and Myeloid) were reprocessed separately for further analysis. PCA was performed using highly variable genes selected for each group, followed by Harmony correction. UMAP projections and clustering were performed using the following number of dimensions and resolution setting for each subset: GG 15 harmony dimensions, clustered with 0.7 resolution, PA 20 dimension, 0.3 resolution, Myeloid, 25 dimensions, 0.3 resolution, T‐cells, 20 dimension, 0.3 resolution.

Differential expression and marker gene identification was performed using findMarkers() from the scran package (v. 1.20.1), with the block argument set to each sample to control for differences in sample compositions in each cluster. The presence of copy number variants (CNVs) was identified using inferCNV.

### 4.5 Cytokine/chemokine analysis

Single‐cell suspensions were thawed in batches and allowed to recover for 24 h in RPMI supplemented with 10% fetal bovine serum and 1% penicillin/streptavidin (R10), at 37°C and 5% CO_2_. To capture a minimum of 50,000 myeloid cells, we used CD45+ microbead isolation (Miltenyi) to eliminate tumor cells. The immune cells were then stimulated with 10 mg/mL lipopolysaccharide for 24 h. Following LPS stimulation, media was collected from the cells and cells were stained with membrane stain supplied in the Human Innate Immune Secretome kit (isoplexis) according to manufacture protocol. Each sample was loaded on to a separate IsoCode chip supplied in the Human Innate Immune Secretome kit (isoplexis) and run on either the IsoSpark. Data were analyzed using IsoSpeak (Isoplexis) software. Media collected from the cells was stored in −80°C. Tumor cells were cultured in R10 for 24 hours in 5%CO_2_ incubator and ultra‐low attachment plate. Tumor conditioned media was harvested by centrifuging and collecting the media supernatant and store in −80°C until used. Cytokine/chemokine concentrations from bulk conditioned media were measured using Milliplex, following manufacturer's instructions as previously described (Supplementary Table 6) [37]. Less than 1% of the immune cells in pLGG are lymphocytes [11] therefore the majority of the cytokines measured will be produced by the LPS stimulated myeloid cells.

### 4.6 Spatial transcriptomic analysis

Frozen samples (*n* = 8) were OCT embedded and sectioned at 10 μm on a Cryostar NX70 cryostat (Thermo Fisher Scientific). Capture sections were fixed with methanol, stained with H&E, and imaged on an Evos M7000 (ThermoFisher) with brightfield settings. Following image capture, capture sections were permeabilized and processed to generate RNA libraries following 10× Visium protocol. Libraries were sequenced to a depth of 70,000 read pairs per spot calculated from the image, on a Novaseq6000 (Illumina) sequencer.

### 4.7 Spatial transcriptomics data analysis

Sequencing data were processed with Space Ranger (10x genomics, v1.2.1), followed by further analysis in R using the Seurat tool suite. Spots were filtered to ensure the number of genes detected between 50 and 15,000, and less than 50% of UMIs mapped to mitochondrial genes. After initial SCTransform normalization on each sample and principal component analysis on merged data, sample integration was performed with Harmony (v0.1.0) using 30 principal components. UMAP dimension reduction and shared nearest neighbor clustering were carried out on 30 principal components, and clustering results at different resolution settings were explored through Clustree (v0.4.3) visualizations.

### 4.8 Deconvolution

Bulk tumor tissue analyzed via Affymetrix array (*n* = 36) was imputed into CYBERSORTx to further explore the single‐cell sequencing findings. This dataset contained 7 GG, 22 PA, and 6 LGG. A CYBERSORTx signature file was generated based on the pLGG scRNA seq data (GSE232316). *P*‐values for the bulk gene expression datasets and the single‐cell derived signature file had a median of 0 (Range 0–0.4). CYBERSORTx analyses were performed in R on absolute mode with 100 permutations using normalized, but not log converted, counts (Mas5.0 normalization for expression array, TPM normalization for RNA‐seq).

### 4.9 Cell–cell communication analysis

Single‐cell RNA sequencing (RNAseq) cell–cell communication analysis was conducted using the “CellChat” R package [38] for inference, analysis, and visualization. CellChat predicts major signaling inputs and outputs for cells and analyzes how these cells and signals coordinate their functions through network analysis and pattern recognition approaches. By leveraging manifold learning and quantitative contrasts, CellChat classifies signaling pathways and delineates both conserved and context‐specific pathways across different datasets. The CellChat package provides a communication probability as a measure of the strength of the Ligand–Receptor interaction. A higher value indicates more interaction strength on average. The analysis was performed independently for each tumor, comparing the myeloid and neoplastic cells from only the same tumor. To summarize the results across all the tumors, we computed the mean_communication_prob. The output has been filtered so that the interactions shown have a *p*‐value of <0.05 in at least 2 tumors (with the n_tumors column indicating the number of tumors with significant hits).

### 4.10 Functional enrichment analyses

Pathway analysis was performed using Metascape (ref). Single‐cell RNA sequencing (RNAseq) Gene Set Enrichment (GSEA) pathway analysis using the Bioconductor “escape” package [39] was performed on the top significantly enriched pathways to compare pathway scores among various neoplastic subclusters. Specifically, analyses were conducted on each cell type in comparison to all other cell types. The analysis utilized the “runEscape” function, applying the “ssGSEA” enrichment score for the individual cells with the selected gene sets and outputting the values as a matrix. These scores facilitate the generation of UMAP scatterplots, which indicate the significance of each interrogated pathway [40].

### 4.11 Statistical analysis

All statistical analyses were performed using R and Prism (GraphPad). For all tests, statistical significance was defined as *P*‐value <0.05. Bonferroni correction was applied to the statistical tests to assess differences in cell type proportions between tumor subtypes.

### 4.12 Study approval

Human samples were collected from surgeries at Children's Hospital Colorado with IRB approval (COM‐IRB 95‐500) (Supplementary Table 1). Written informed consent was received prior to participation.

## AUTHOR CONTRIBUTIONS

SZ and JML designed the project; KR, TL, and RF performed bioinformatics analyses; SZ wrote the initial manuscript; SZ, AD, and AG prepared samples for analyses; AD and AG assisted with design and interpretation of spatial transcriptomic and cytokine analyses respectively; TH and MR provided samples; KR, TL, RF, SZ, AG, AD prepared and reviewed figures; JML, TF, JG, MC, JD, JR, MG, EB, AG, AD, RV, NW, NF, KR participated in data and manuscript review and edit.

## FUNDING INFORMATION

This study was supported by grants from the Peter Barton Family Fund for Clinical and Translational Cancer Research at The Children's Hospital Colorado Foundation, Alex's Lemonade Stand Foundation, Amazon Goes Gold for Kids with Cancer, University of Colorado Cancer Center Support Grant (P30CA046934), NIH/NCATS Colorado CTSA Grant (UM1 TR004399), Olivia Caldwell Foundation, and the Morgan Adams Foundations.

## CONFLICT OF INTEREST STATEMENT

The authors declare no competing interests.

## Supporting information


**Data S1.** Supporting Information.

**Data S2.** Figures.

**Supplementary Table 1.** Summary of pLGG sample characteristics.

**Supplementary Table 2:** The number of cells comprising each neoplastic subclusters following scRNA‐seq analysis are shown.

**Supplementary Table 3:** The number of cells comprising each non‐neoplastic subclusters from following scRNA‐seq analysis are shown.

**Supplementary Table 4:** PA spatial transcriptomics marker genes.

**Supplementary Table 5:** GG spatial transcriptomics marker genes.

**Supplemental Table 6:** Experimental details and cell counts for single cell cytokine assay.

## Data Availability

ScRNA‐seq and ST data have been deposited in the National Center for Biotechnology Information Gene Expression Omnibus (GEO) database and are publicly accessible through GEO SuperSeries accession number GSE232316 (https://www.ncbi.nlm.nih.gov/geo/query/acc.cgi?acc=GSE232316). Analysis code will be provided in a github repository upon publication.
