# Supplementary material for: Multi‐pronged analysis of pediatric low‐grade glioma and ganglioglioma reveals a unique tumor microenvironment associated with BRAF alterations
Source: Brain Pathol. 2025 Jun 30;35(6):e70023. doi: 10.1111/bpa.70023 (PMC12488260; doi:10.1111/bpa.70023)
Supplement: Supplementary file 1 — Data S1. Supporting Information. [file BPA-35-e70023-s006.docx]

**Supplementary Methods**

*BRAF mutation detection using single-cell RNA sequencing (scRNA-seq) data*

BRAF V600E detection:

1. We used scRNA-seq FASTQ files and processed them with Cell Ranger v7.1.0 using the cellranger count pipeline, which generates the corresponding BAM files for each sample.
2. To visually inspect the presence of mutations, BAM files were loaded into IGV Integrative Genomics Viewer) using the Human GRCh38/hg38 reference genome. We focused on the BRAF V600E mutation region, located at chr7:140753330–140753340.
3. Next, we used bcftools to perform variant calling at this locus.

BRAF:KIAA 1549 detection:

1. We used STAR (v2.7.10a) to map the scRNA-seq FASTQ files to the GRCh38 human genome using the following reference files:

- Genome FASTA: GRCh38.primary_assembly.genome.fa
- Gene annotation GTF: gencode.v46.annotation.gtf

1. For mapping, chimeric read detection was enabled with STAR to capture potential fusion events, generating sorted BAM files.
2. We then used Arriba (v2.5.0) to detect gene fusions from the STAR BAM output, using the following resources (provided by Arriba package):

- Known fusions list: known_fusions_hg38_GRCh38_v2.5.0.tsv.gz
- Blacklist of false-positive fusions: blacklist_hg38_GRCh38_v2.5.0.tsv.gz
- Protein domain annotations: protein_domains_hg38_GRCh38_v2.5.0.gff3

*BRAF mutation detection using RNA using bulk RNA sequencing data*

BRAF V600E detection:

FASTQ files were converted to BAM files at an earlier time. The same process was followed as the one when scRNA seq data was used.

BRAF:KIAA 1549 detection:

We used bulk FASTQ files instead of single cell FASTQ files. All the steps and methods are the same as scRNA seq methods with stricter fusion detection settings.
