# Supplementary material for: Multi‐pronged analysis of pediatric low‐grade glioma and ganglioglioma reveals a unique tumor microenvironment associated with BRAF alterations
Source: Brain Pathol. 2025 Jun 30;35(6):e70023. doi: 10.1111/bpa.70023 (PMC12488260; doi:10.1111/bpa.70023)
Supplement: Supplementary file 2 — Data S2. Figures. [file BPA-35-e70023-s004.pptx]

## Slide 1
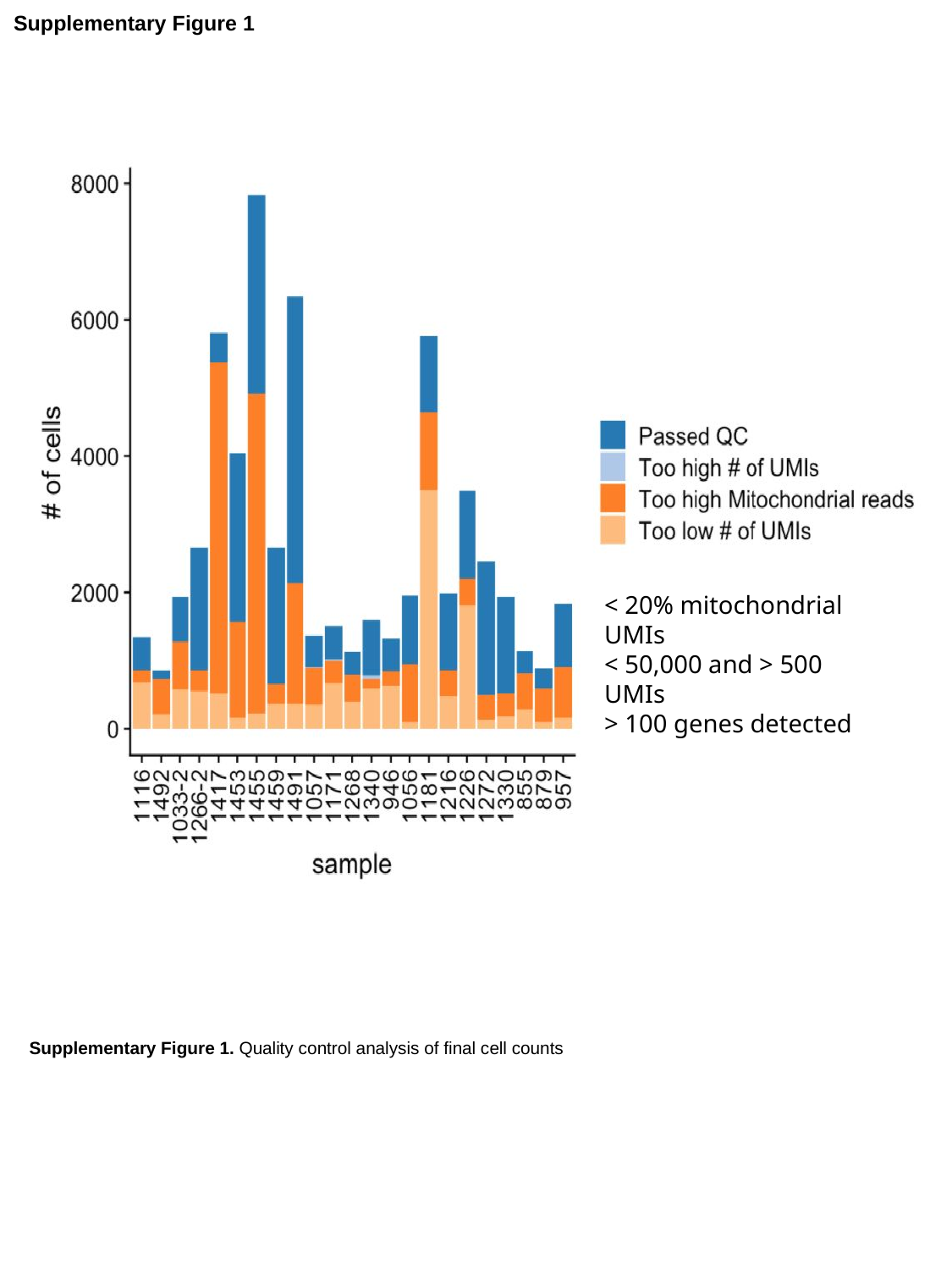

Supplementary Figure 1
< 20% mitochondrial UMIs
< 50,000 and > 500 UMIs
> 100 genes detected
Supplementary Figure 1. Quality control analysis of final cell counts

## Slide 2
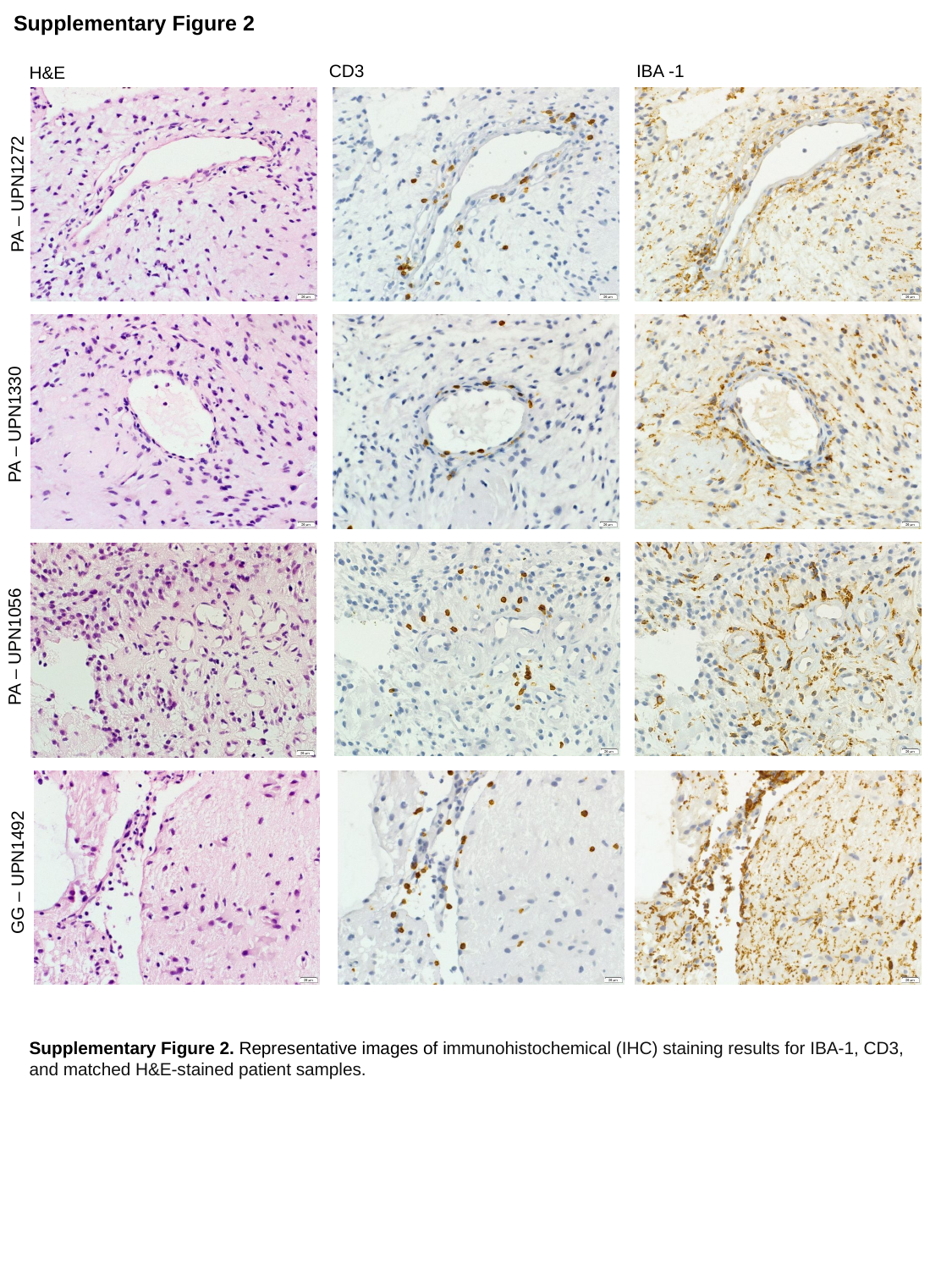

Supplementary Figure 2
CD3
IBA -1
H&E
 PA – UPN1272
 PA – UPN1330
 PA – UPN1056
 GG – UPN1492
Supplementary Figure 2. Representative images of immunohistochemical (IHC) staining results for IBA-1, CD3, and matched H&E-stained patient samples.

## Slide 3
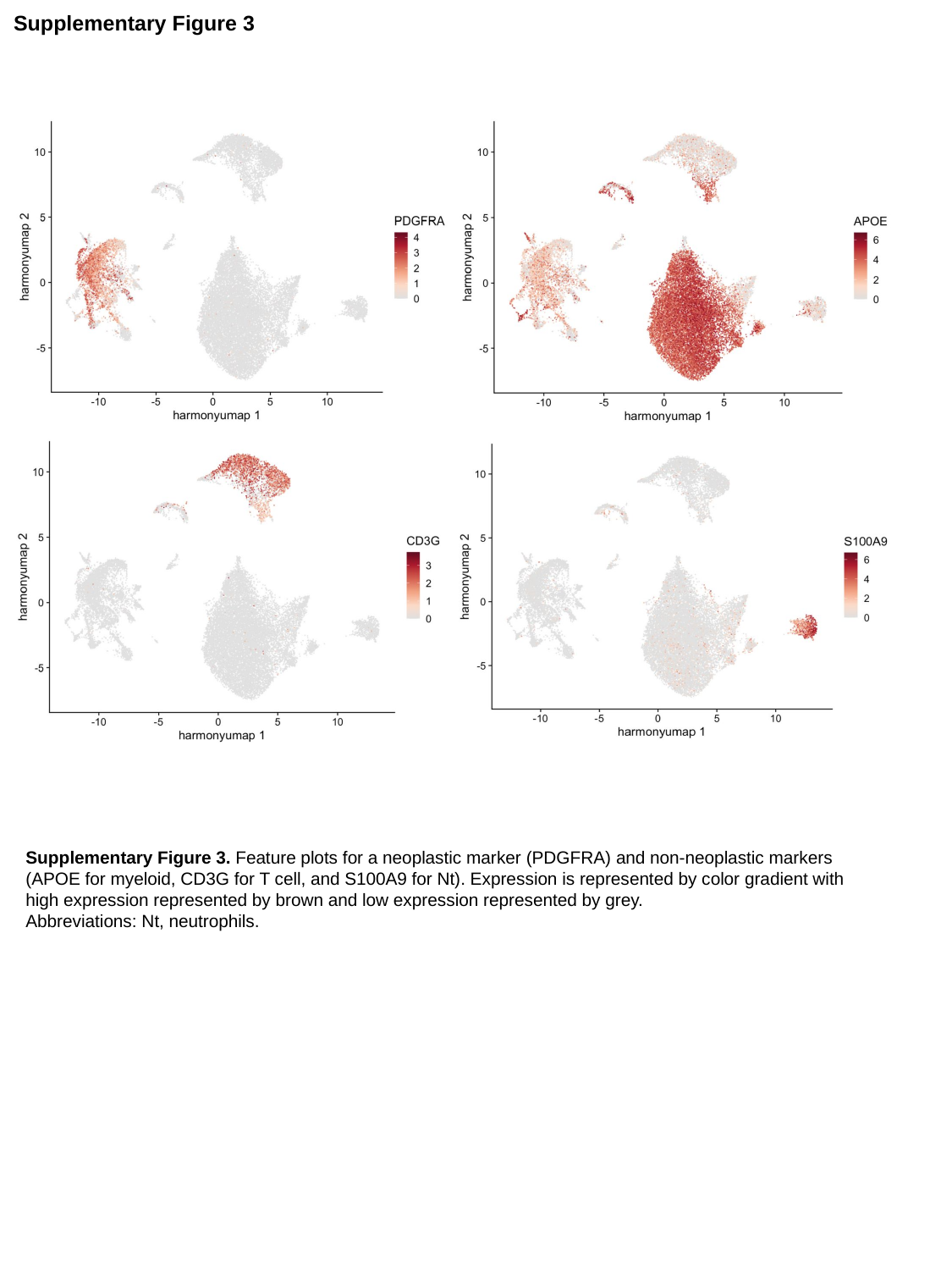

Supplementary Figure 3
Supplementary Figure 3. Feature plots for a neoplastic marker (PDGFRA) and non-neoplastic markers (APOE for myeloid, CD3G for T cell, and S100A9 for Nt). Expression is represented by color gradient with high expression represented by brown and low expression represented by grey.
Abbreviations: Nt, neutrophils.

## Slide 4
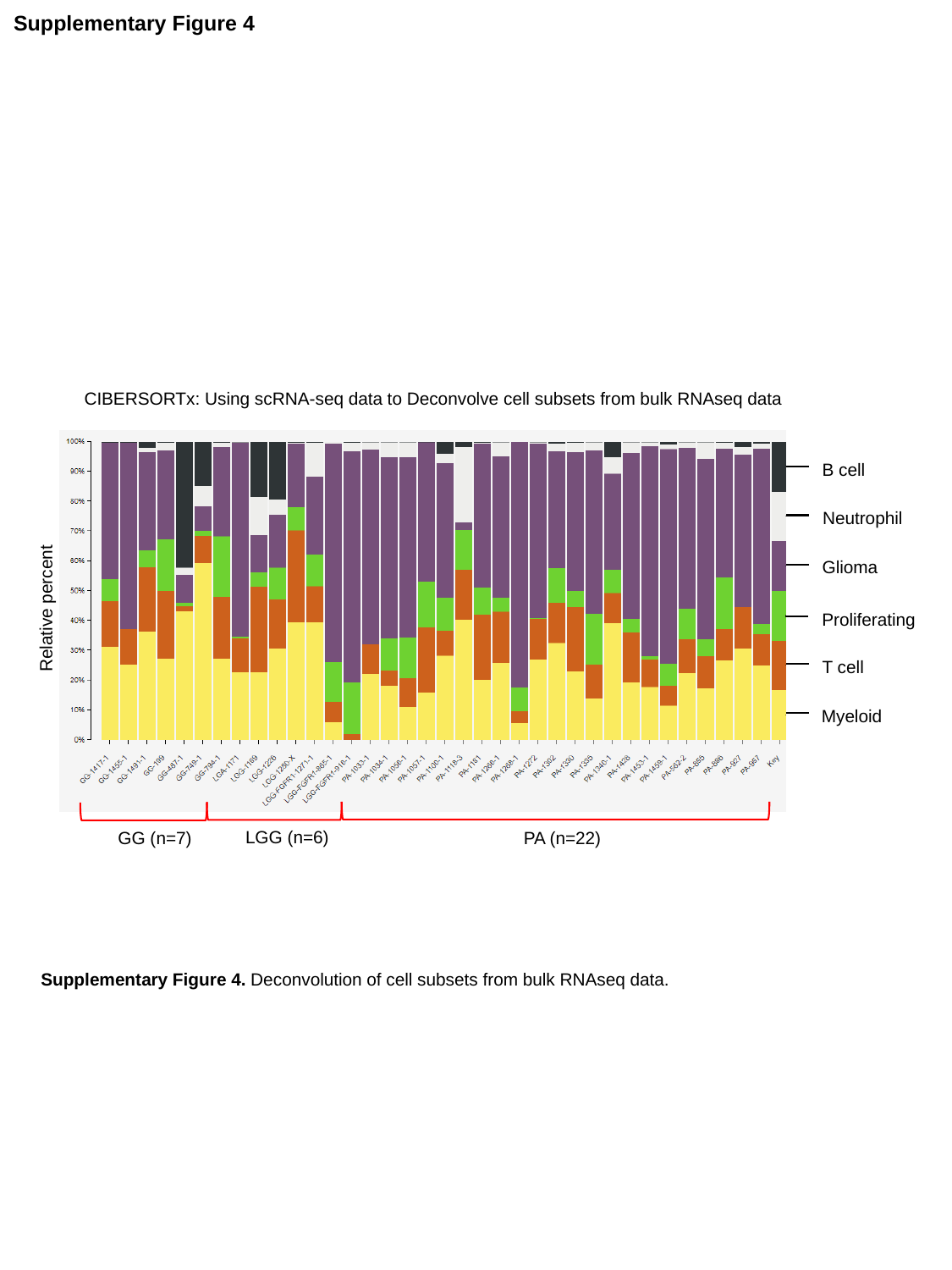

Supplementary Figure 4
CIBERSORTx: Using scRNA-seq data to Deconvolve cell subsets from bulk RNAseq data
B cell
Neutrophil
Glioma
Relative percent
Proliferating
T cell
Myeloid
LGG (n=6)
GG (n=7)
PA (n=22)
Supplementary Figure 4. Deconvolution of cell subsets from bulk RNAseq data.

## Slide 5
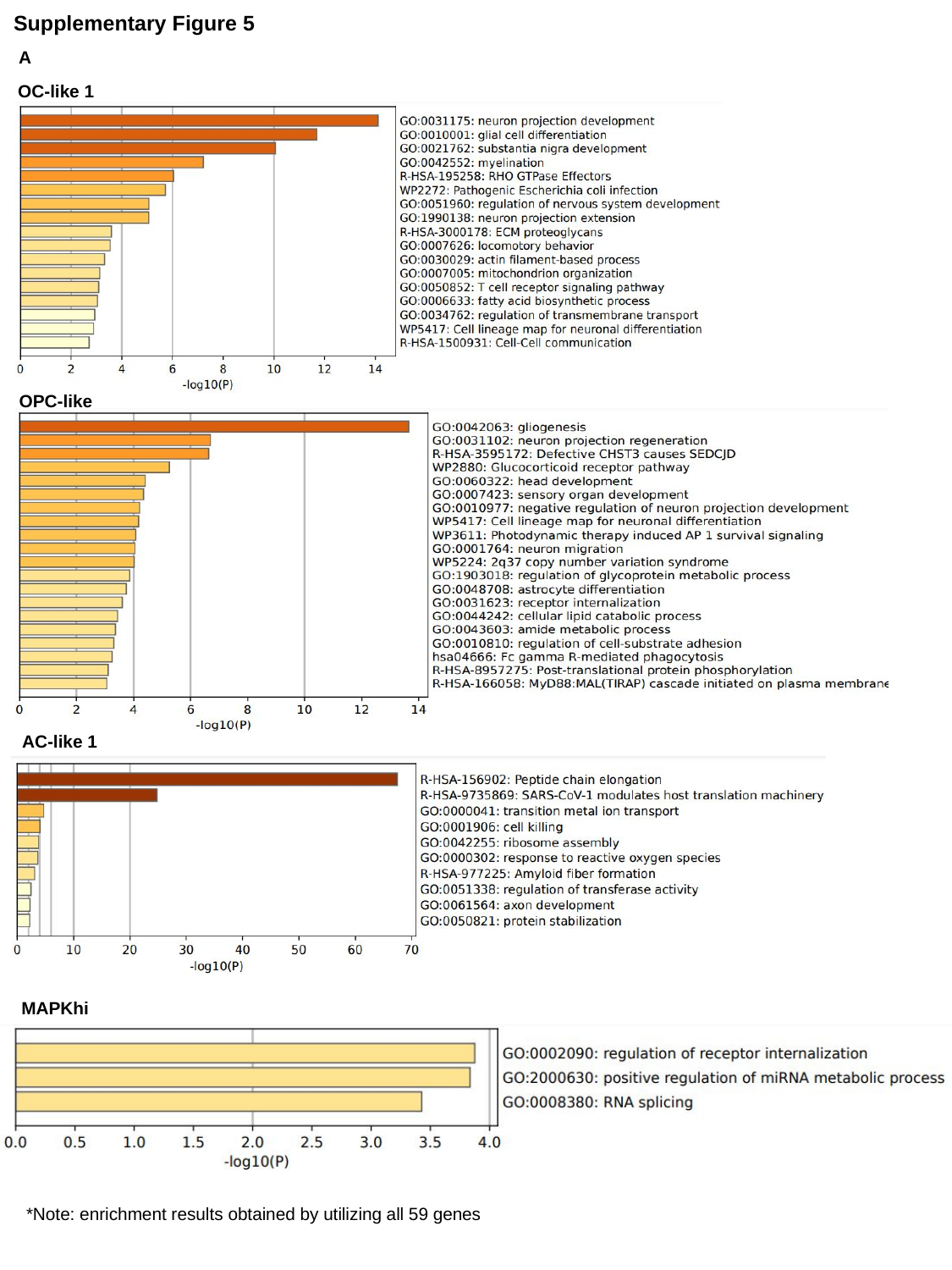

Supplementary Figure 5
A
OC-like 1
OPC-like
AC-like 1
MAPKhi
*Note: enrichment results obtained by utilizing all 59 genes

## Slide 6
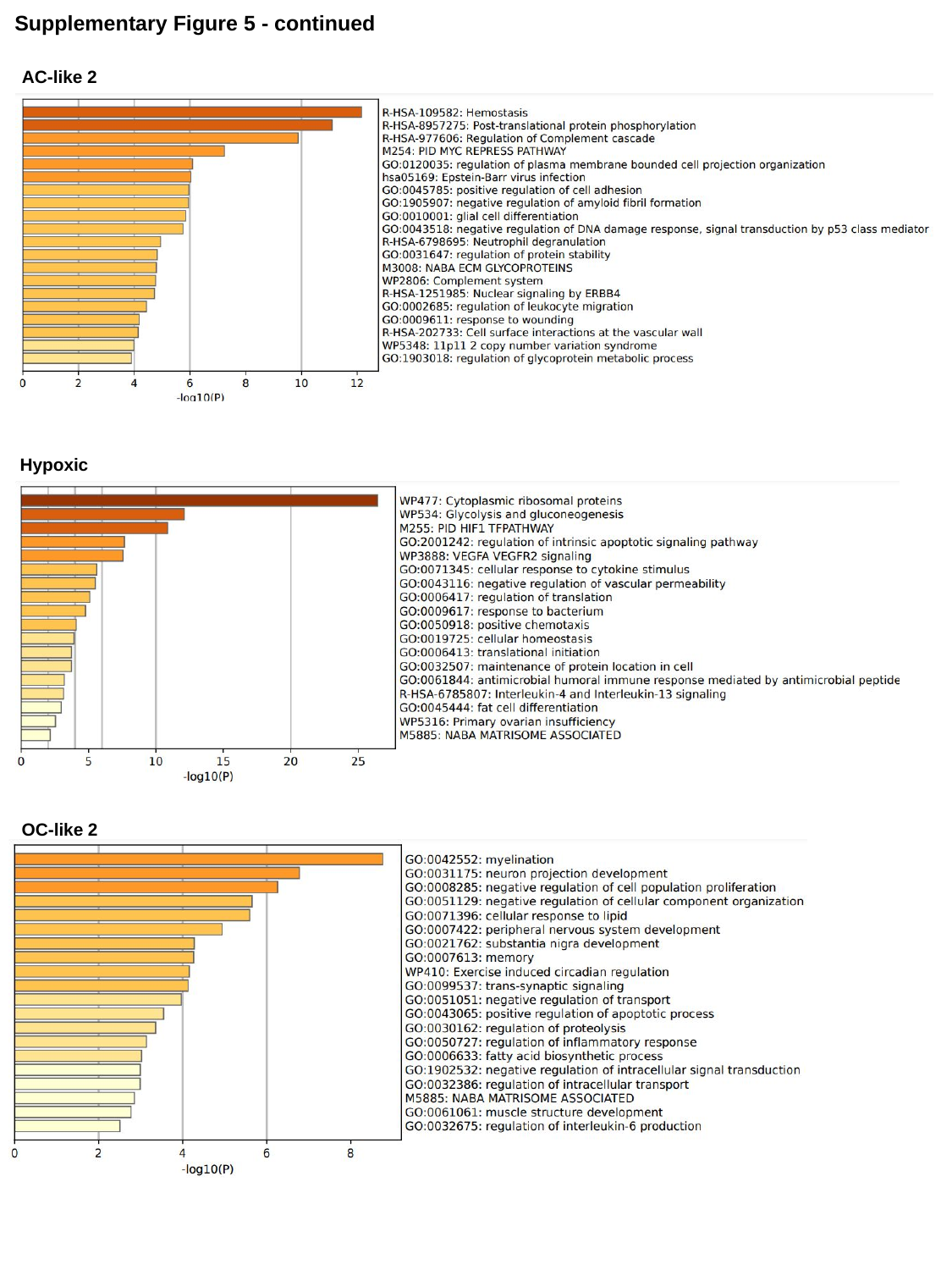

Supplementary Figure 5 - continued
AC-like 2
Hypoxic
OC-like 2

## Slide 7
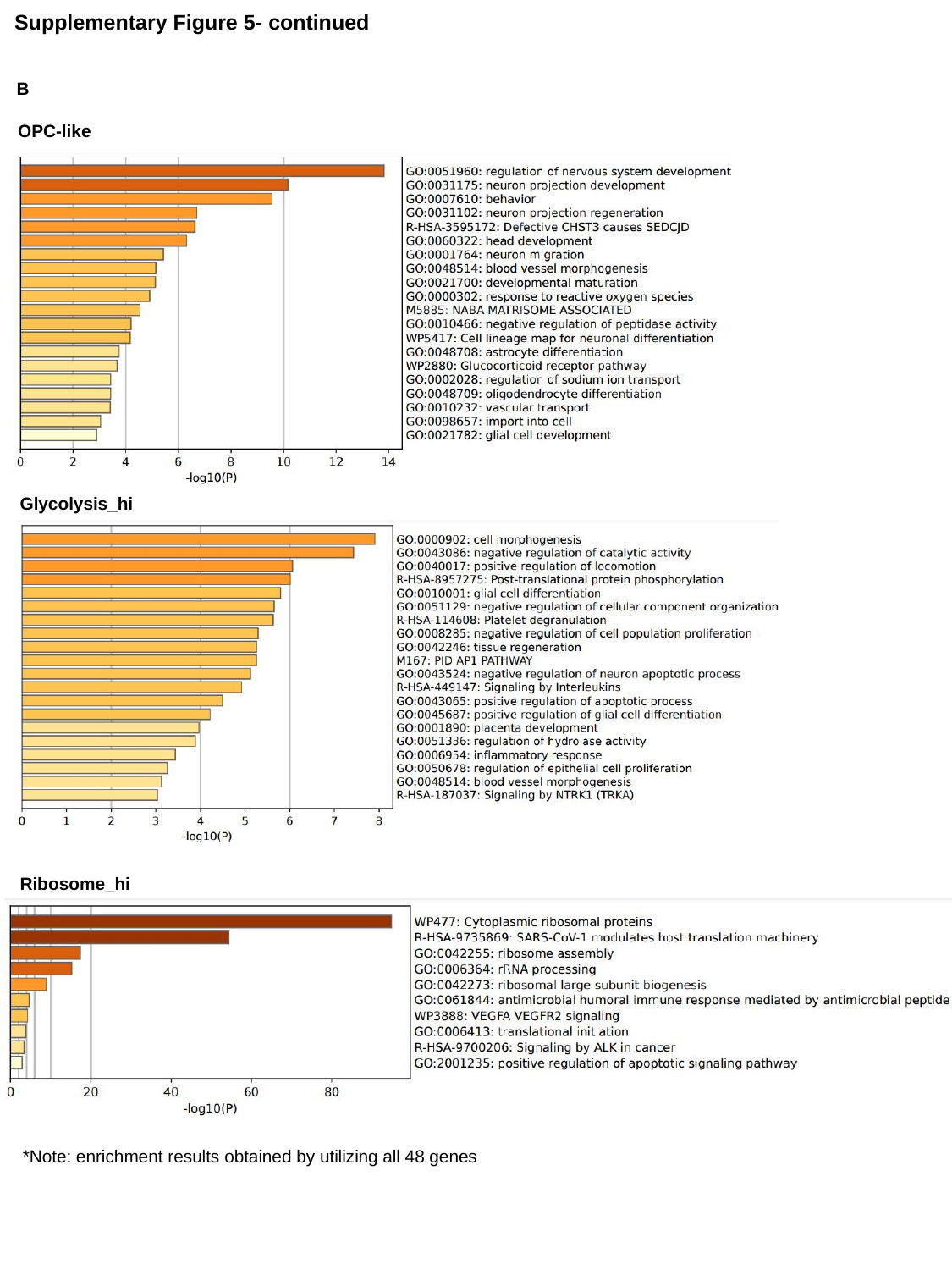

Supplementary Figure 5- continued
B
OPC-like
Glycolysis_hi
Ribosome_hi
*Note: enrichment results obtained by utilizing all 48 genes

## Slide 8
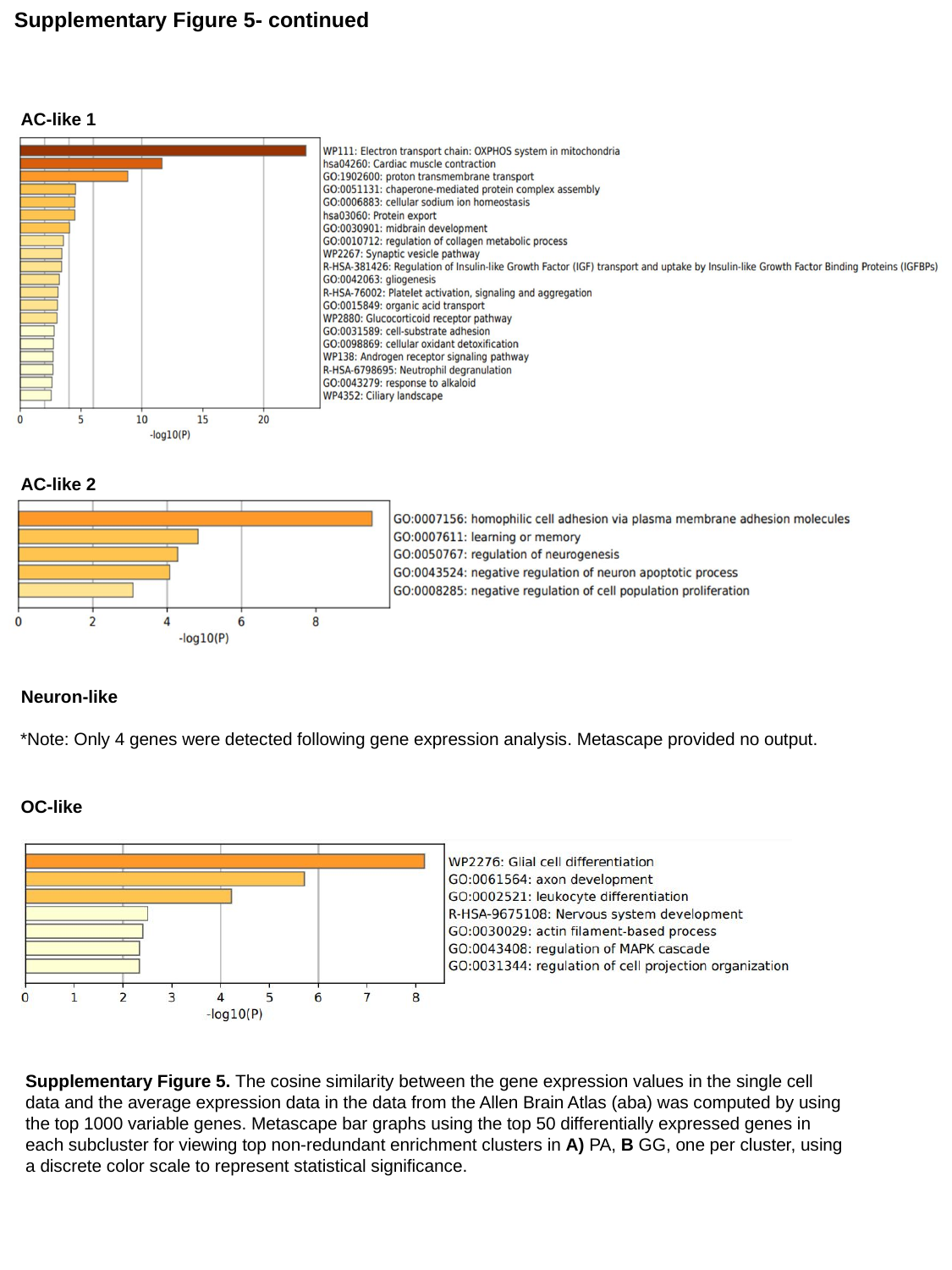

Supplementary Figure 5- continued
AC-like 1
AC-like 2
Neuron-like
*Note: Only 4 genes were detected following gene expression analysis. Metascape provided no output.
OC-like
Supplementary Figure 5. The cosine similarity between the gene expression values in the single cell data and the average expression data in the data from the Allen Brain Atlas (aba) was computed by using the top 1000 variable genes. Metascape bar graphs using the top 50 differentially expressed genes in each subcluster for viewing top non-redundant enrichment clusters in A) PA, B GG, one per cluster, using a discrete color scale to represent statistical significance.

## Slide 9
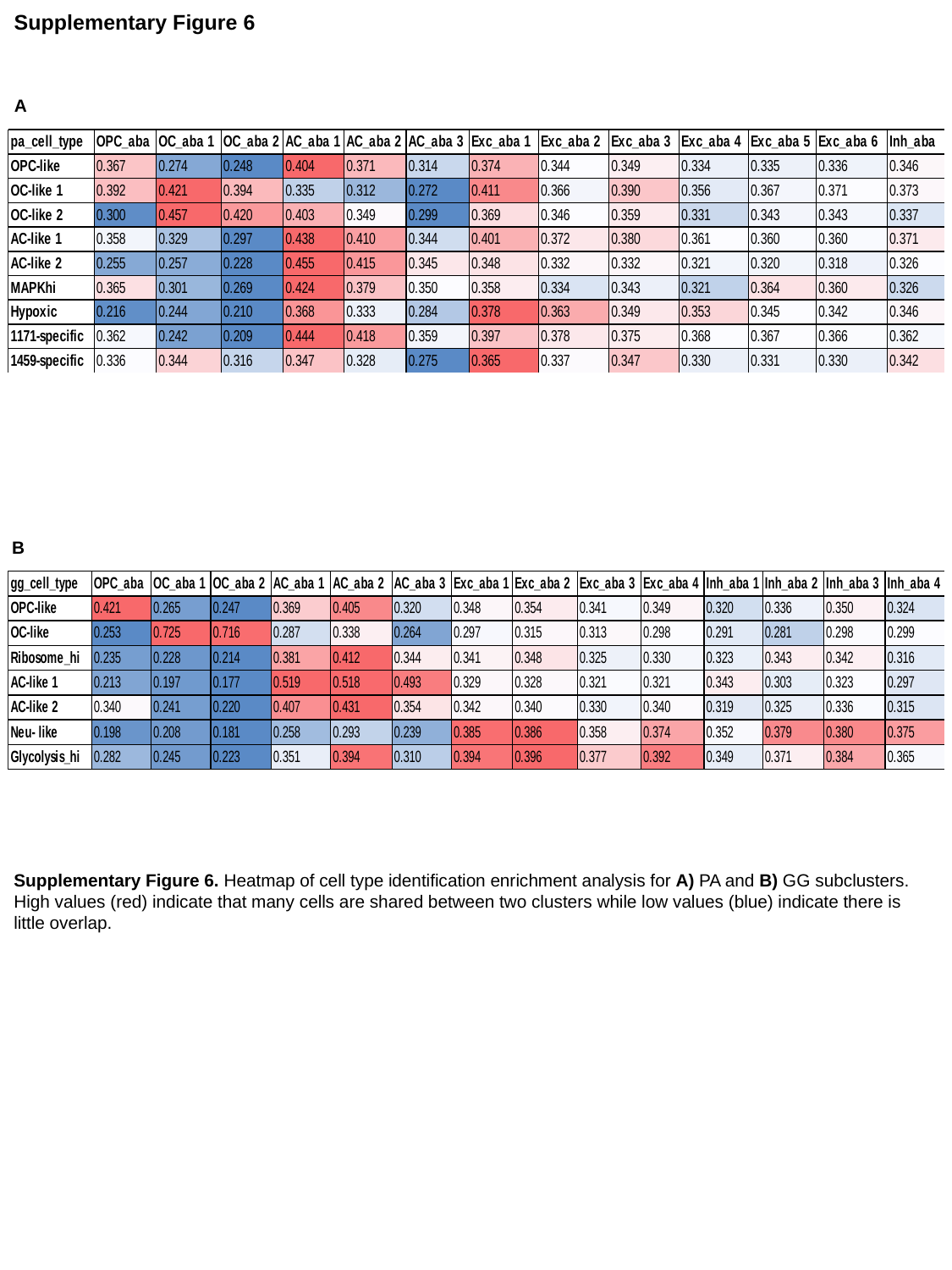

Supplementary Figure 6
A
B
Supplementary Figure 6. Heatmap of cell type identification enrichment analysis for A) PA and B) GG subclusters. High values (red) indicate that many cells are shared between two clusters while low values (blue) indicate there is little overlap.

## Slide 10
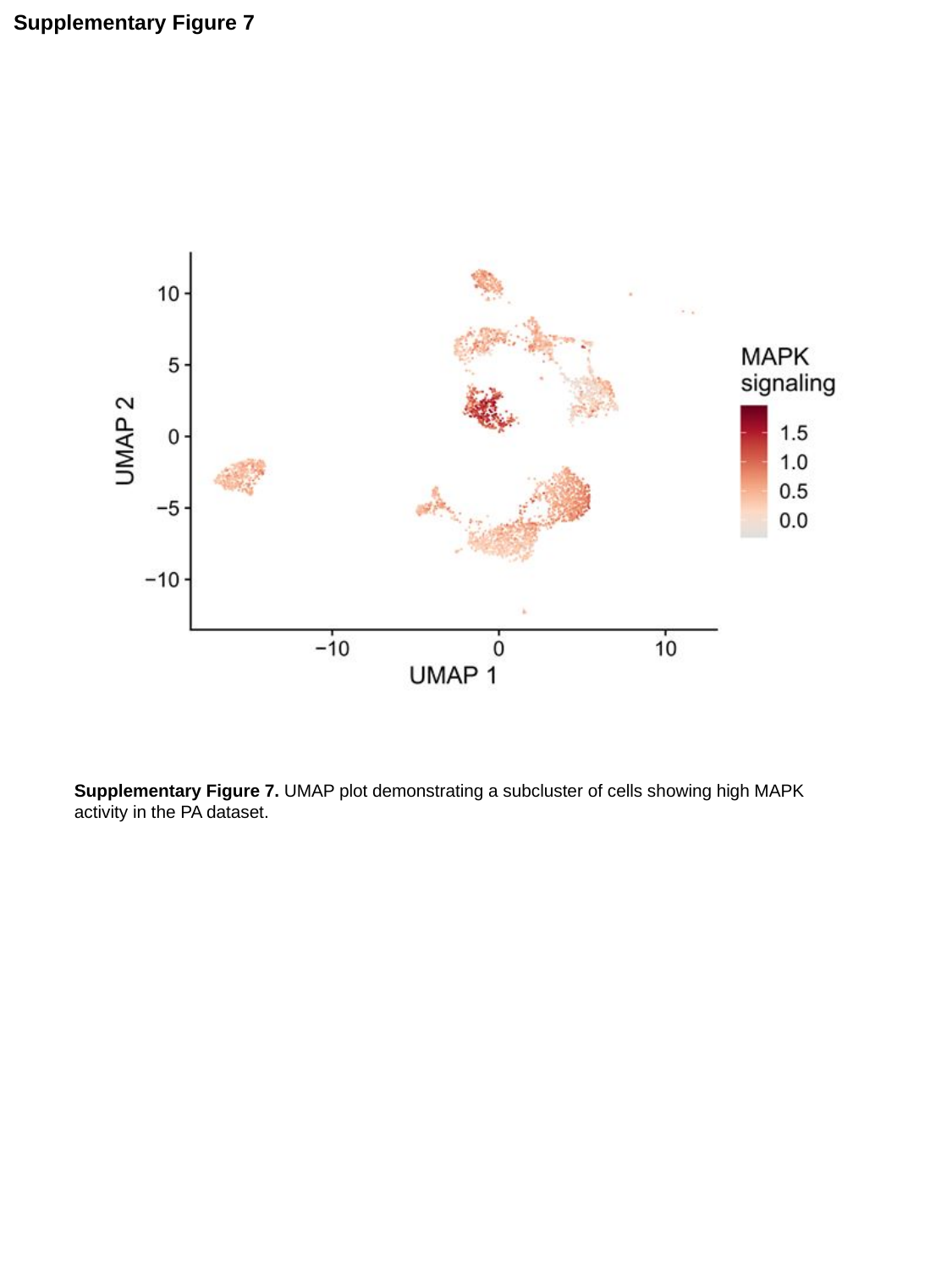

Supplementary Figure 7
Supplementary Figure 7. UMAP plot demonstrating a subcluster of cells showing high MAPK activity in the PA dataset.

## Slide 11
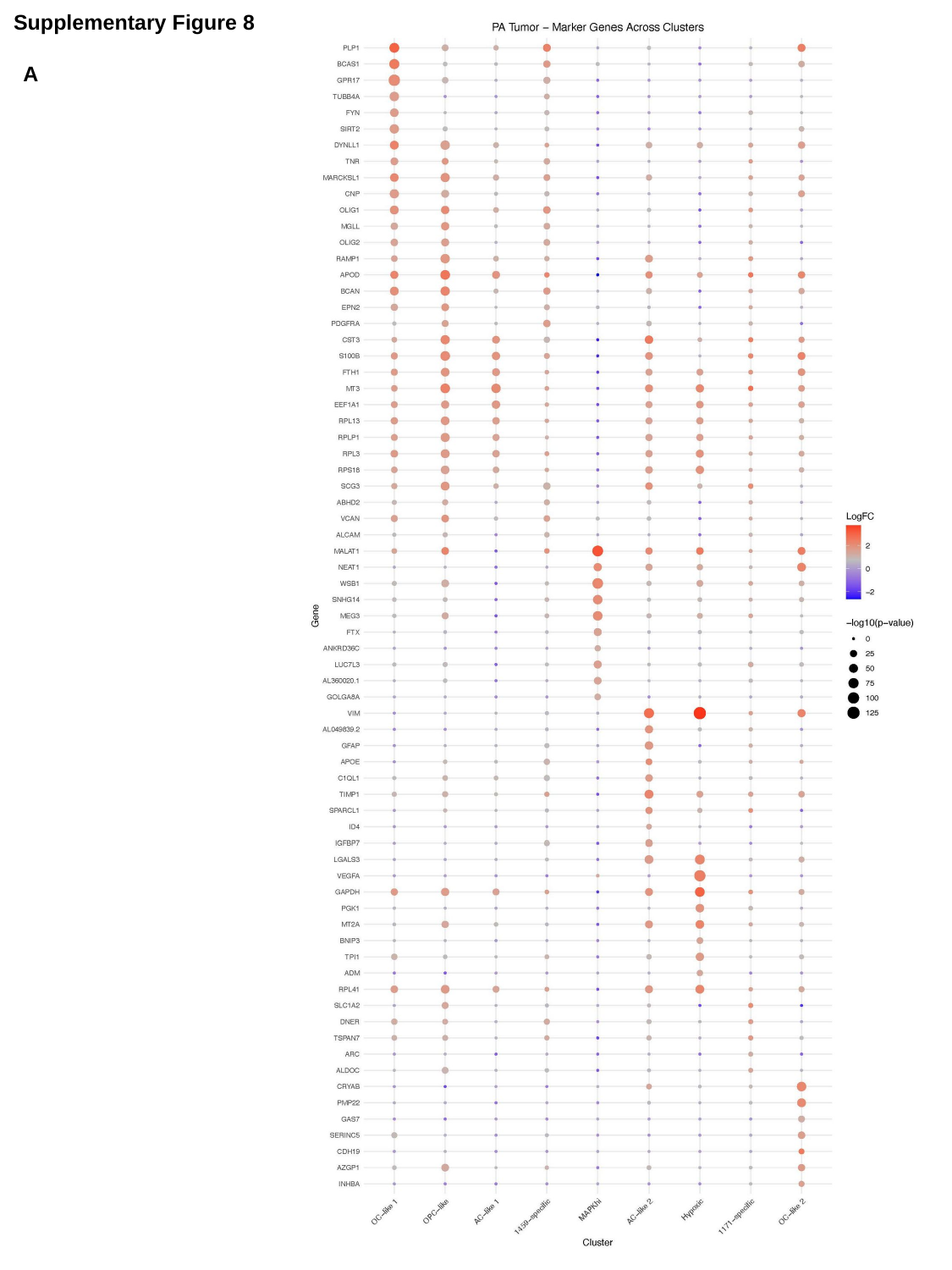

Supplementary Figure 8
A

## Slide 12
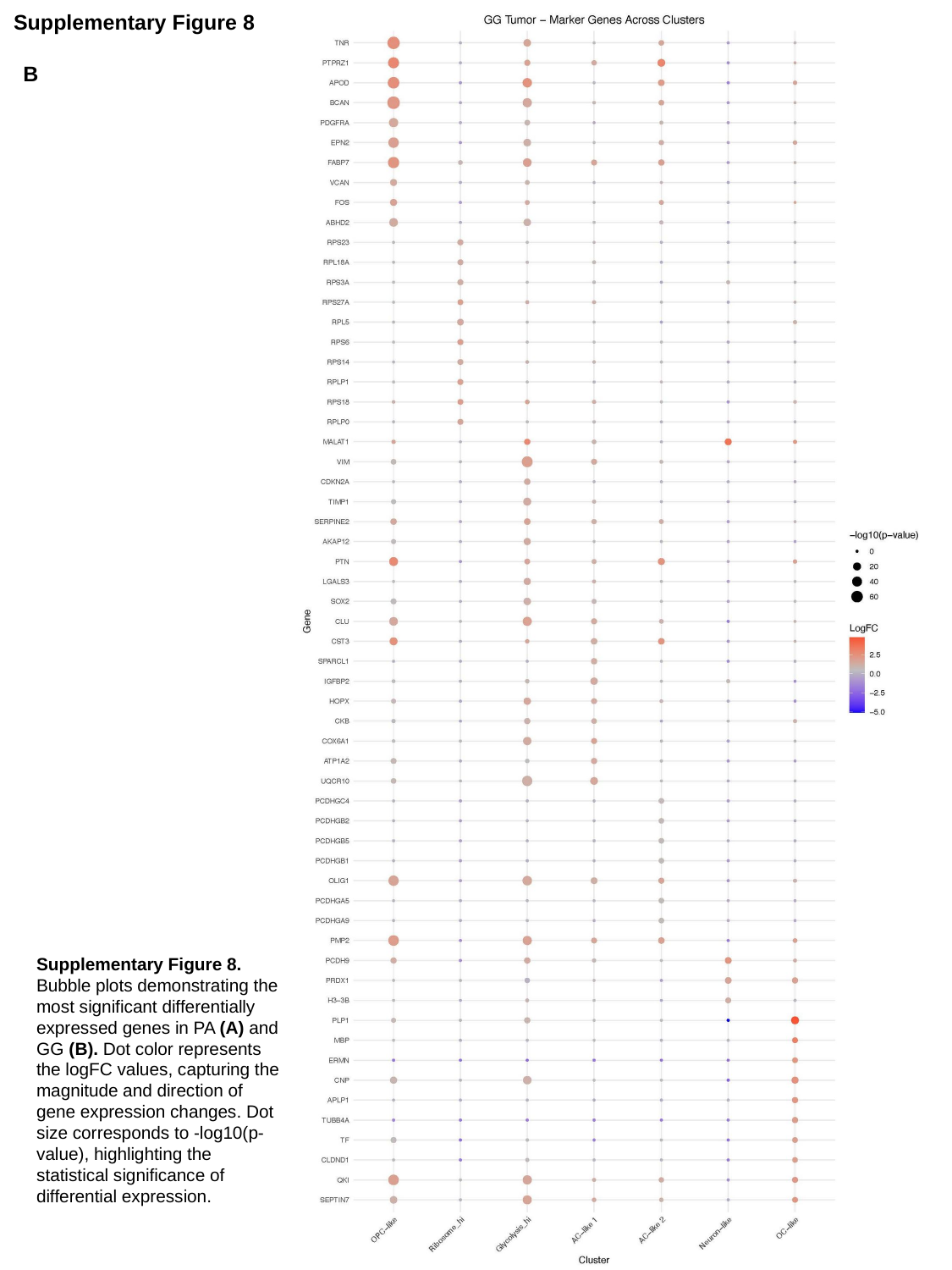

Supplementary Figure 8
B
Supplementary Figure 8. Bubble plots demonstrating the most significant differentially expressed genes in PA (A) and GG (B). Dot color represents the logFC values, capturing the magnitude and direction of gene expression changes. Dot size corresponds to -log10(p-value), highlighting the statistical significance of differential expression.

## Slide 13
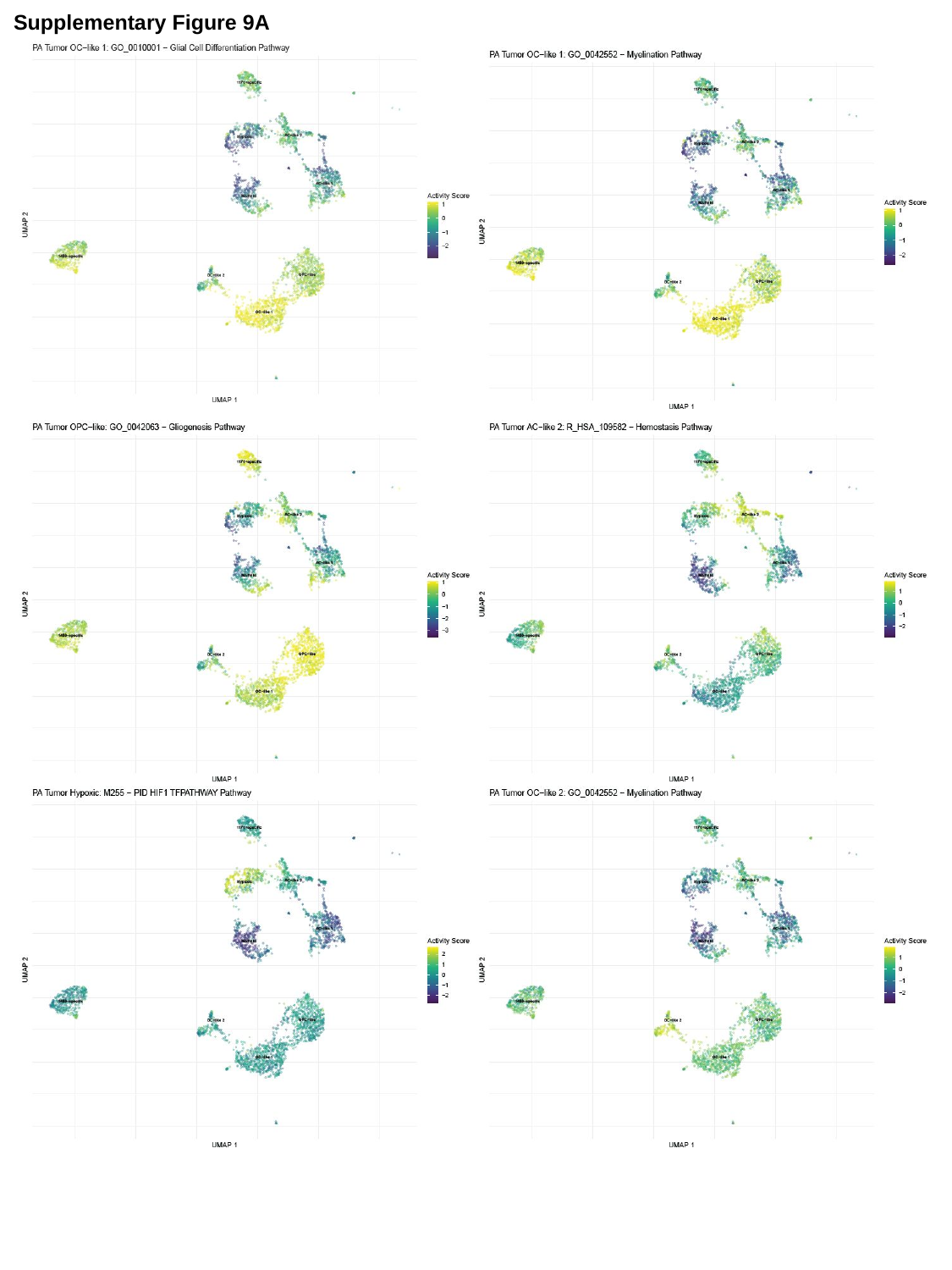

Supplementary Figure 9A

## Slide 14
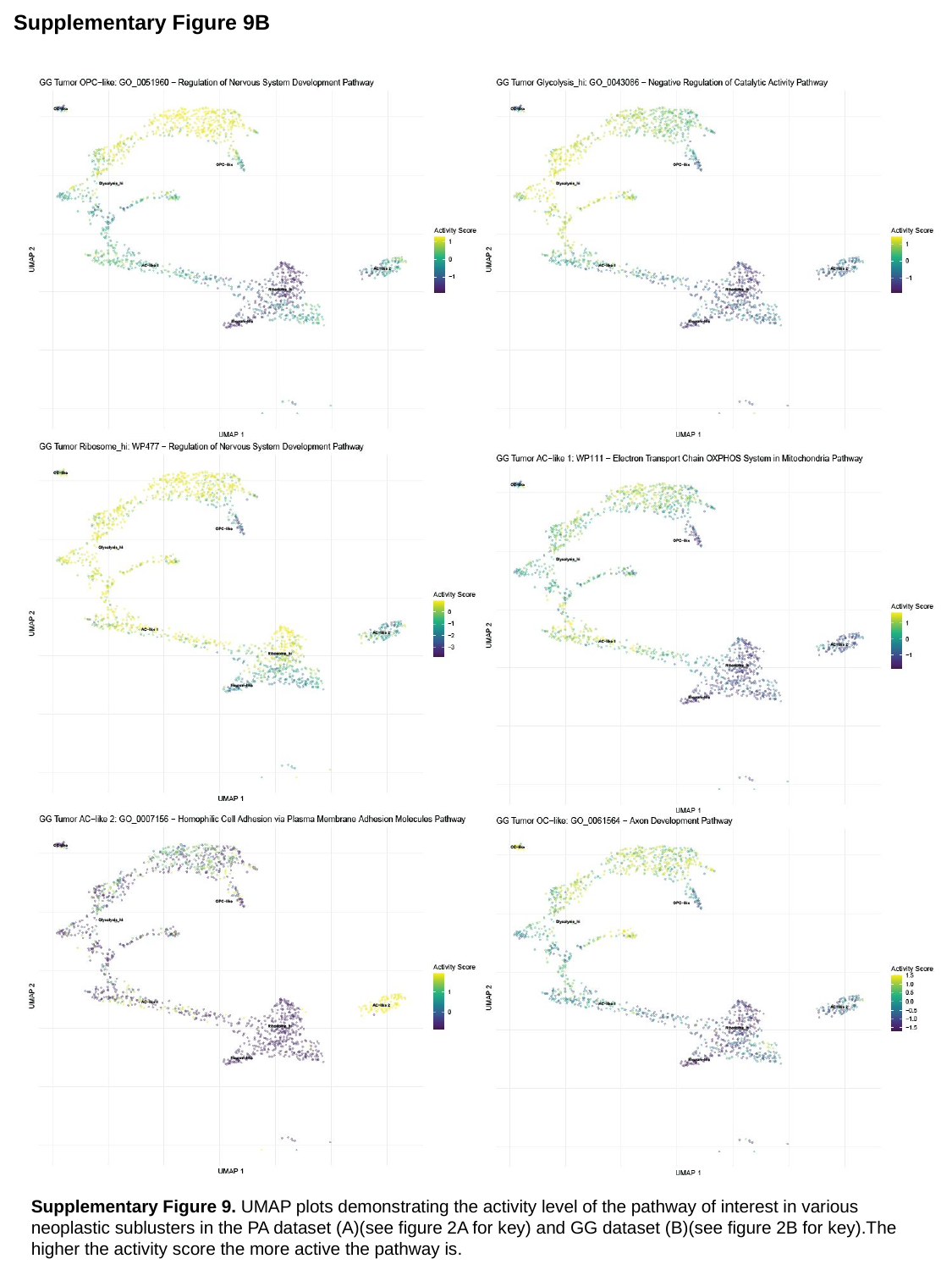

Supplementary Figure 9B
Supplementary Figure 9. UMAP plots demonstrating the activity level of the pathway of interest in various neoplastic sublusters in the PA dataset (A)(see figure 2A for key) and GG dataset (B)(see figure 2B for key).The higher the activity score the more active the pathway is.

## Slide 15
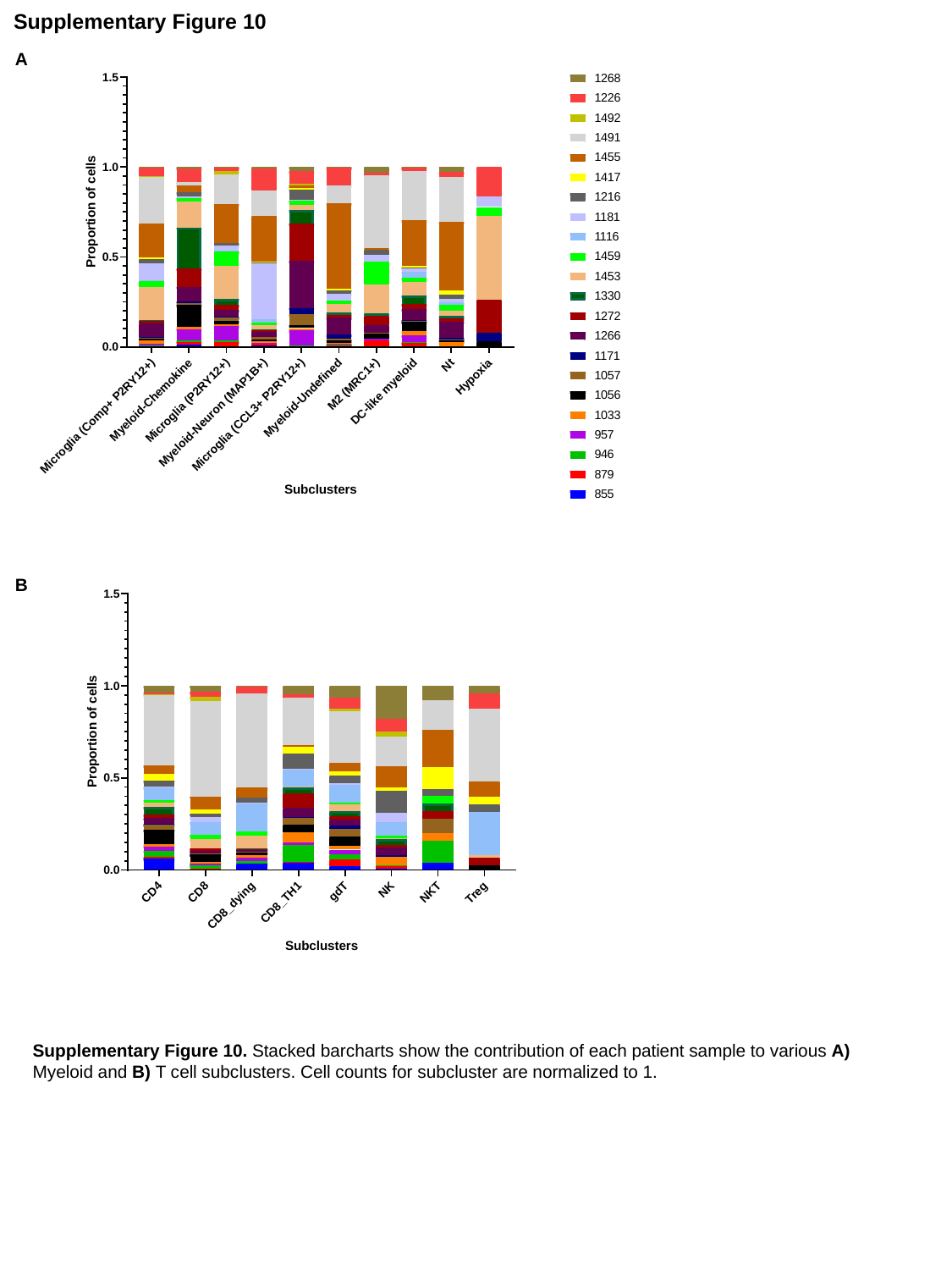

Supplementary Figure 10
A
B
Supplementary Figure 10. Stacked barcharts show the contribution of each patient sample to various A) Myeloid and B) T cell subclusters. Cell counts for subcluster are normalized to 1.

## Slide 16
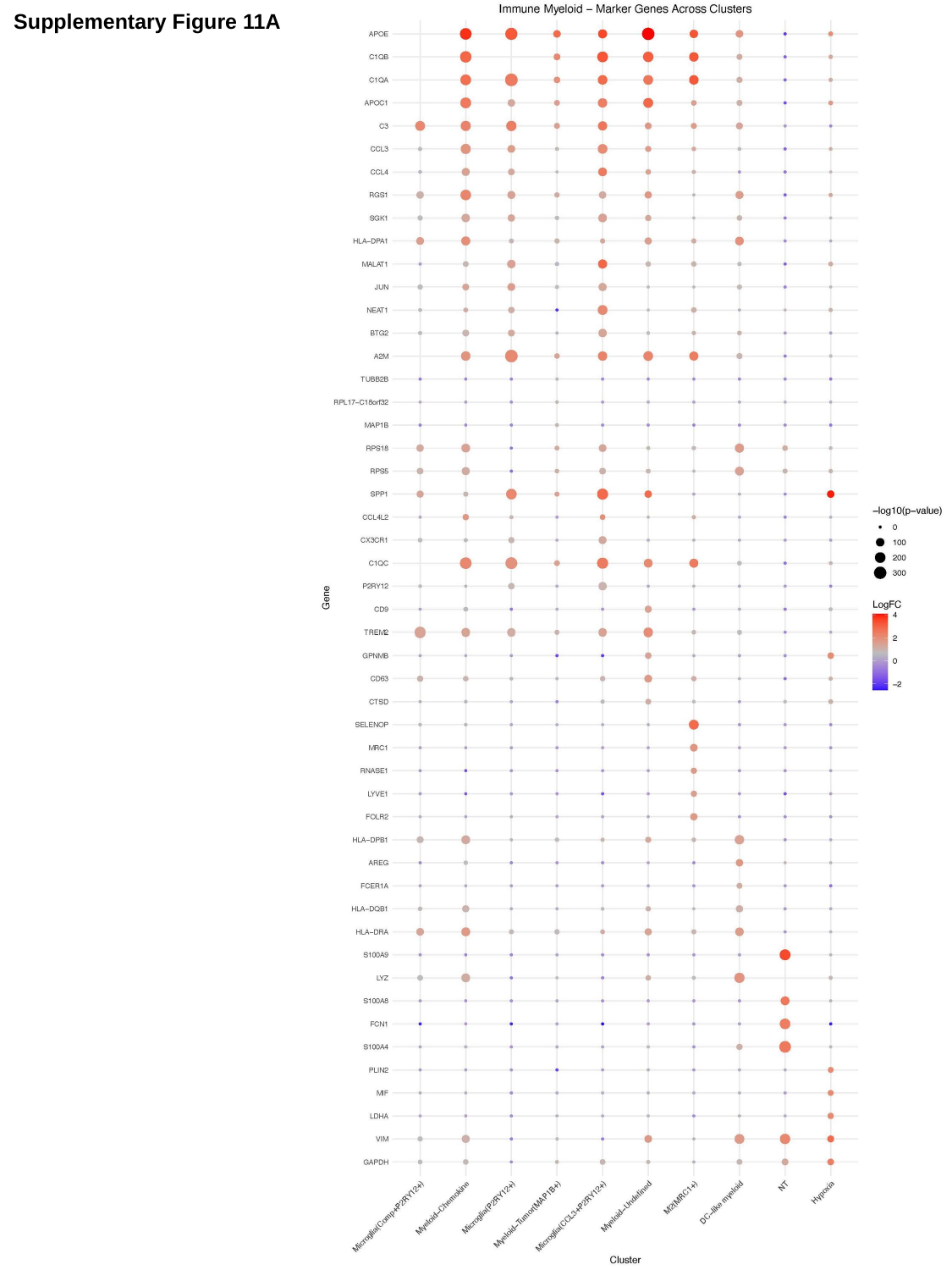

Supplementary Figure 11A

## Slide 17
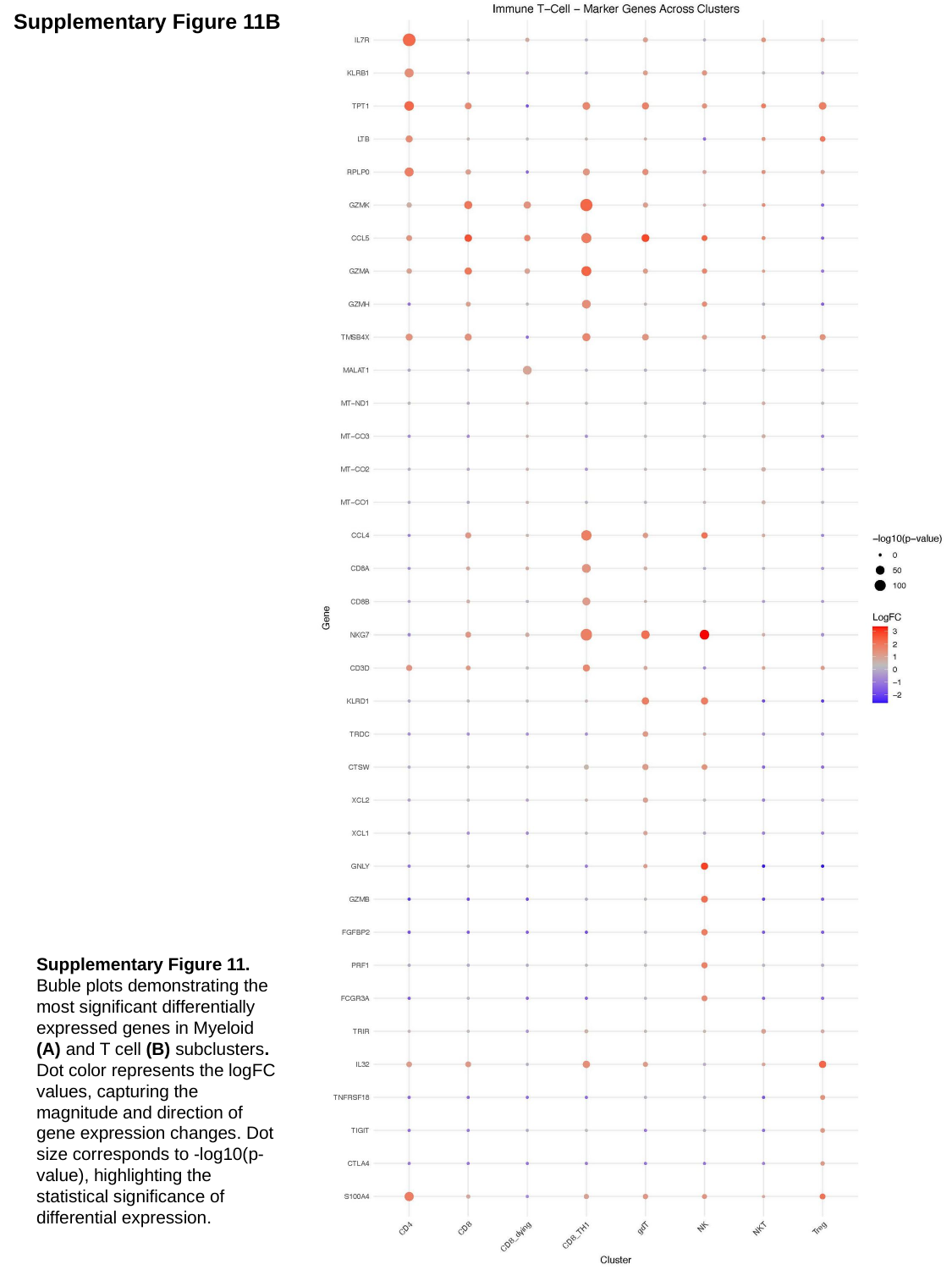

Supplementary Figure 11B
Supplementary Figure 11. Buble plots demonstrating the most significant differentially expressed genes in Myeloid (A) and T cell (B) subclusters. Dot color represents the logFC values, capturing the magnitude and direction of gene expression changes. Dot size corresponds to -log10(p-value), highlighting the statistical significance of differential expression.

## Slide 18
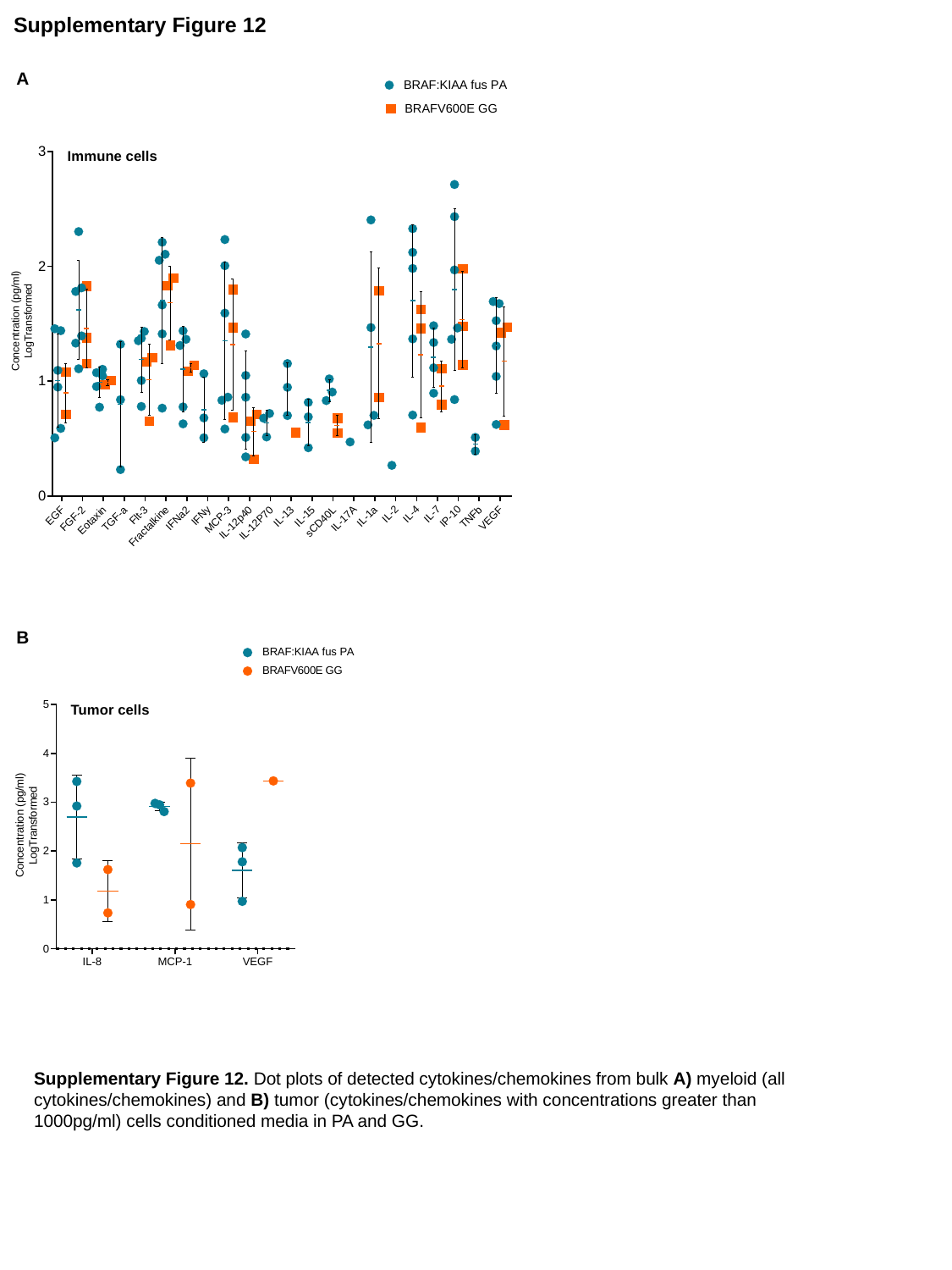

Supplementary Figure 12
A
Immune cells
B
Tumor cells
Supplementary Figure 12. Dot plots of detected cytokines/chemokines from bulk A) myeloid (all cytokines/chemokines) and B) tumor (cytokines/chemokines with concentrations greater than 1000pg/ml) cells conditioned media in PA and GG.

## Slide 19
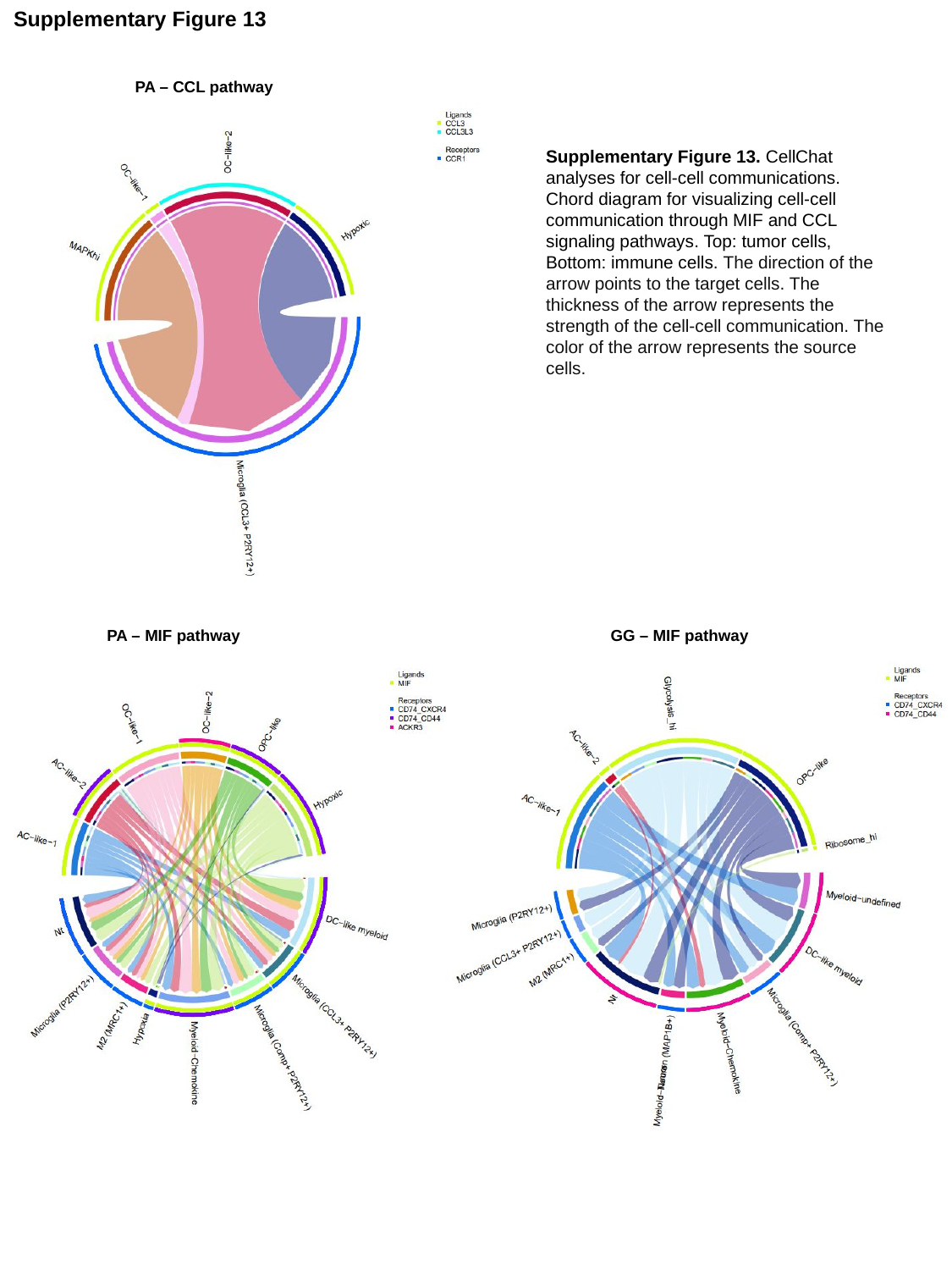

Supplementary Figure 13
PA – CCL pathway
Supplementary Figure 13. CellChat analyses for cell-cell communications. Chord diagram for visualizing cell-cell communication through MIF and CCL signaling pathways. Top: tumor cells, Bottom: immune cells. The direction of the arrow points to the target cells. The thickness of the arrow represents the strength of the cell-cell communication. The color of the arrow represents the source cells.
PA – MIF pathway
GG – MIF pathway
Tumor

## Slide 20
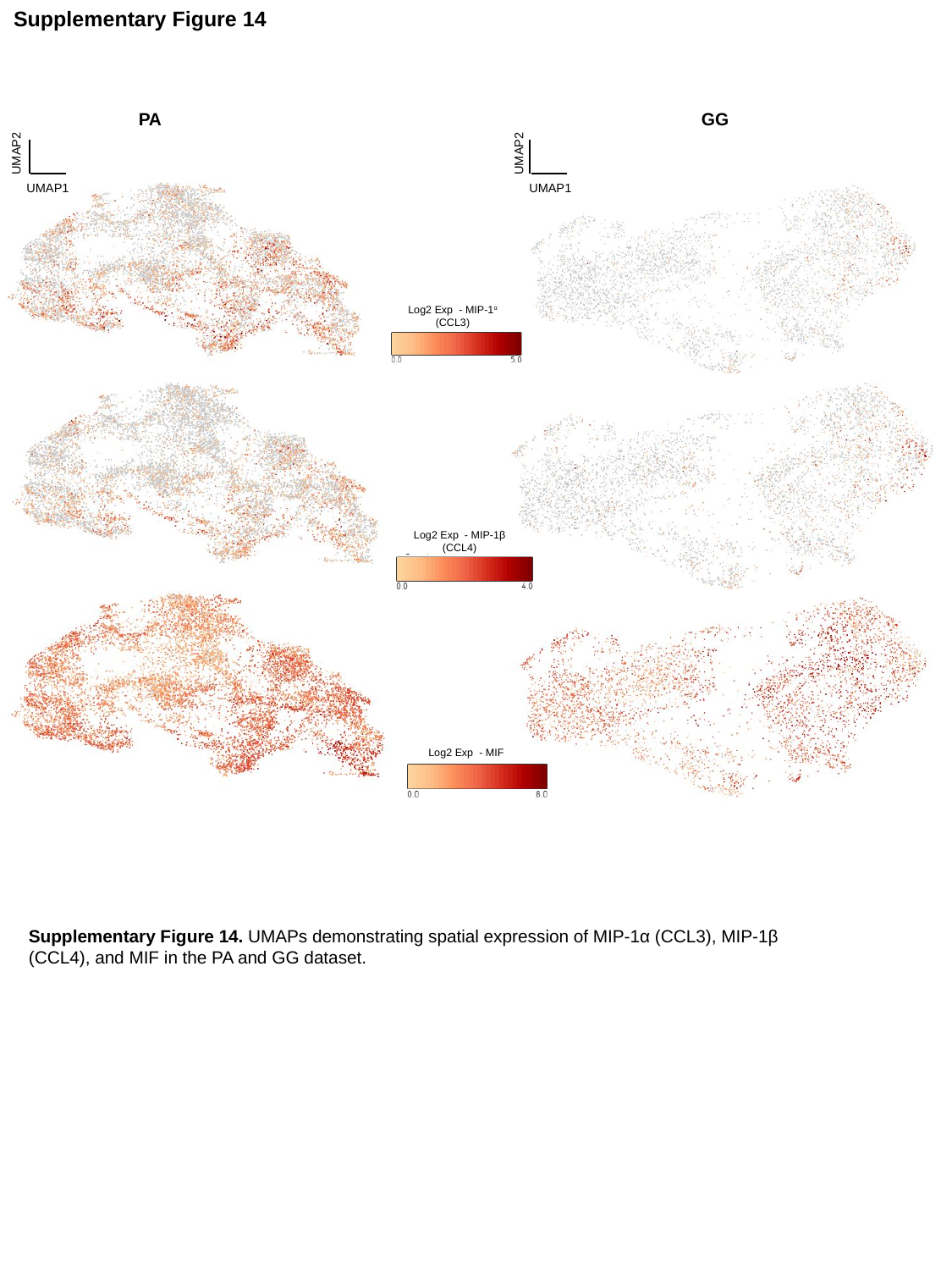

Supplementary Figure 14
PA
GG
UMAP2
UMAP2
UMAP1
UMAP1
Log2 Exp - MIP-1α
(CCL3)
Log2 Exp - MIP-1β
(CCL4)
Log2 Exp - MIF
Supplementary Figure 14. UMAPs demonstrating spatial expression of MIP-1α (CCL3), MIP-1β (CCL4), and MIF in the PA and GG dataset.
